# Real-time single-molecule 3D tracking in *E. coli* based on cross-entropy minimization

**DOI:** 10.1038/s41467-023-36879-1

**Published:** 2023-03-11

**Authors:** Elias Amselem, Bo Broadwater, Tora Hävermark, Magnus Johansson, Johan Elf

**Affiliations:** Department of Cell and Molecular Biology, Science for Life Laboratory, Uppsala University, Husarg. 3, SE-75124 Uppsala, Sweden

**Keywords:** Single-molecule biophysics, Super-resolution microscopy, Fluorescence spectroscopy

## Abstract

Reaching sub-millisecond 3D tracking of individual molecules in living cells would enable direct measurements of diffusion-limited macromolecular interactions under physiological conditions. Here, we present a 3D tracking principle that approaches the relevant regime. The method is based on the true excitation point spread function and cross-entropy minimization for position localization of moving fluorescent reporters. Tests on beads moving on a stage reaches 67 nm lateral and 109 nm axial precision with a time resolution of 0.84 ms at a photon count rate of 60 kHz; the measurements agree with the theoretical and simulated predictions. Our implementation also features a method for microsecond 3D PSF positioning and an estimator for diffusion analysis of tracking data. Finally, we successfully apply these methods to track the Trigger Factor protein in living bacterial cells. Overall, our results show that while it is possible to reach sub-millisecond live-cell single-molecule tracking, it is still hard to resolve state transitions based on diffusivity at this time scale.

## Abstract (web summary)

Single-molecule 3D tracking is critical to understand macromolecular dynamics but achieving this at a sub-millisecond resolution remains challenging. Here the authors present a 3D tracking method based on cross-entropy minimization and the true excitation point spread function.

## Introduction

Advances in microscopy and nanoscopy are approaching the temporal and spatial scale where intracellular biochemistry occurs. Single-molecule tracking is a critical technique to study intracellular kinetics by analyzing changes in diffusivity^1^ without perturbing the cells^2^. Recently, improved single-molecule localization of reporters with a small photon budget was demonstrated by several labs, addressing a key limitation in live-cell single-molecule tracking^3–16^. They achieved enhanced localization precision by exciting the fluorophores with structured optical beams and comparing the modulations in emitted fluorescence to the excitation patterns. The principle, referred to as modulation-enhanced localization (SM-MEL), is different from traditional single-molecule localization techniques such as PALM/STORM^17,18^ where the emitted point spread function (PSF) is used for localization. Under the umbrella of SM-MEL, several strategies have been demonstrated, each optimized for a different purpose. The camera-based versions like ROSE^3^, SIMPLE^4^, SIMFLUX^5^, and ModLoc^6^ target a large field of view. The imaging-oriented scanning counterparts, MINFLUX^7,8^ and p-MINFLUX^9^, aim for an optimal resolution with a minimal photon budget per fluorophore localization assuming slow-moving/stationary reporters. Tracking-oriented systems like Orbital scanning^10–12,19^, SMCT–FCS^13^, 3D-DyPLoT^14,15^, 3D-SMART^20^, and TSUNAMI^16^ target spectroscopic and dynamical processes. Although these strategies have very different implementations, they all localize reporters using the SM-MEL principle under various assumptions.

Both the camera and the minimal photon flux strategies have shown great performance in imaging. When the reporter is moving slowly^7,13,21,22^ or is confined^8,9^ within a small volume, an information-optimized localization method can be used. Only a few well-placed excitation shots are then needed to obtain a unique photon signature that pinpoints the position at high temporal and spatial resolution in a well-defined and confined region. However, for single-molecule tracking, these techniques have not yet been demonstrated for the observation of fast 3D dynamical processes in a cellular structure. The main limitation of the camera systems is the frame rate. For the scanning counterpart, a common problem is that fast molecules move out of the limited region over which the resolution is optimized, see Supplementary Note 1.

For fast reporters moving in a larger volume, a more responsive and/or larger tracking volume is required. Having a small resolution-optimized pattern together with a highly responsive system pose high requirements on hardware bandwidth, shot noise, and photophysics of the reporter. A large illumination pattern, on the other hand, has a negative impact on time and spatial resolution. Thus, a balance between system response and illumination pattern size is required when tracking reporters with a large dynamic range and there will be a tradeoff between time and spatial resolution affecting both slow and fast-moving reporters.

Here, we describe a generalized theoretical treatment of the SM-MEL principle which we also implement in a 3D single-molecule tracking system. Our implementation is based on minimizing the cross-entropy between the emitted photon counts and the true excitation PSF. It introduces a regularization schema that stabilizes the maximum a posteriori (MAP) estimate of the position when tracking reporters using a large illumination pattern. The large pattern effectively reduces the bandwidth requirements of the piezo positioning system, which is often limiting when tracking fast reporters. On the more practical side, we have introduced a method which removes the need for fast short-range axial scanning equipment. In contrast to the common approach where a Tunable Acoustic Gradient (TAG) lens is used for axial capability, we have introduced axial capability by encoding z information into an engineered PSF, which is only moved in 2D. We tested the microscope and our understanding of the system by moving and tracking fluorescently labeled 20*nm* beads and comparing the results to our simulation of the tracking system. Next, we used simulations to evaluate our capability of tracking molecules in cellular geometries and inferring their binding kinetics using a diffusion point estimator in conjunction with a hidden Markov model (HMM). Finally, the microscope was unleashed on single molecules moving in living bacterial cells.

## Results

### Single-molecule tracking concept

Consider the problem of estimating the unknown position **r**_*p*_ = (*x*_*p*_, *y*_*p*_, *z*_*p*_) of a diffusing, fluorescently labeled molecule. The approach adopted here is based on structured illumination together with Bayesian statistics and priors, which makes it possible to extend the localization problem of stationary and slow-moving reporters to more rapidly moving ones. Our labeled molecule is excited by a sequence of known point spread functions (PSF), Φ_*i*_(**r**) indexed by the subscript *i*. The emitted photons, *n*_*i*_, are detected with a single-photon counting avalanche photodiode (SPC-APD). In general, the PSFs are arbitrary and include Gaussians, doughnuts, or even random speckle patterns that are experimentally measured. But, for simplicity, we assume that the same PSF, Φ_*i*_ = Φ(**r** − **r**_*i*_), is used at the positions, **r**_*i*_. The excitation and measurement times are considered to be long compared to the fluorescence relaxation time and the emitted photons can thus be assumed to follow a Poisson distribution with a mean *λ*_*i*_. Also, we assume that the mean photon counts are proportional to the excitation PSF intensity. Thus *λ*_*i*_ = *c* ⋅ Φ(**r**_*p*_ − **r**_*i*_) where *c* can be considered a fluorophore photon efficiency, a combination of excitation, emission, and detection efficiency per excitation power. The likelihood, given *m* PSF shots at locations ${\{{{{{{{{{\bf{r}}}}}}}}}_{i}\}}_{i=1}^{m}$, to obtain the photon counts ${\{{n}_{i}\}}_{i=1}^{m}$ from a fluorophore at **r**_*p*_ with a photon efficiency *c* can now be stated as

1 $$L(\{{n}_{i}\}|{{{{{{{{\bf{r}}}}}}}}}_{p},c,\,\{{{{{{{{{\bf{r}}}}}}}}}_{i}\})={\prod }_{i}\frac{1}{{n}_{i}!}{e}^{-{\lambda }_{i}}{\lambda }_{i}^{{n}_{i}}=\left({\prod }_{i}\frac{1}{{n}_{i}!}\right)\left({e}^{{\sum }_{i}-c\cdot {{\Phi }}({{{{{{{{\bf{r}}}}}}}}}_{p}-{{{{{{{{\bf{r}}}}}}}}}_{i})+{n}_{i}\ln (c\cdot {{\Phi }}({{{{{{{{\bf{r}}}}}}}}}_{p}-{{{{{{{{\bf{r}}}}}}}}}_{i}))}\right)$$

Using Bayes rule we can invert the problem to obtain the likelihood, *L*(**r**, *c*∣{*n*_*i*_}, {**r**_*i*_}), that the fluorophore is at the position **r** with photon efficiency *c* given the photon counts ${\{{n}_{i}\}}_{i=1}^{m}$ and PSF positions ${\{{{{{{{{{\bf{r}}}}}}}}}_{i}\}}_{i=1}^{m}$. The unknown parameter *c* can be estimated by following the standard maximum likelihood procedure

2 $$\tilde{c}({{{{{{{\bf{r}}}}}}}})=\frac{{\sum }_{i}{n}_{i}}{{\sum }_{i}{{\Phi }}({{{{{{{{\bf{r}}}}}}}}}_{i}-{{{{{{{\bf{r}}}}}}}})}$$

where $\tilde{}$ will be used to indicate estimators. The estimator is simply the ratio of the total number of photons detected to the total PSF intensity and gives a map of the estimates of fluorophore photon efficiency over all allowed positions **r**. Inserting Eq. (2) back into the likelihood function Eq. (1) with some simplifications gives

3 $$L({{{{{{{\bf{r}}}}}}}}|\{{n}_{i}\},\{{{{{{{{{\bf{r}}}}}}}}}_{i}\})=C\cdot {\left(\mathop{\prod}\limits_{i}{\left(\frac{{{{\Phi }}}_{i}}{{\sum }_{i}{{{\Phi }}}_{i}}\right)}^{\frac{{n}_{i}}{{\sum }_{i}{n}_{i}}}\right)}^{{\sum }_{i}{n}_{i}}$$

where *C*({*n*_*i*_}) is a scaling factor that depends only on {*n*_*i*_} and thus does not affect the overall shape of the likelihood landscape, and Φ_*i*_ depends on both **r** and **r**_*i*_. It should be noted that the negative log of Eq. (3) is up to an additive constant equal to the cross-entropy between the photon counts and the PSFs. Derivation of Eq. (3) without passing through Eq. (2) is also possible by the multinomial approach^7–9^ or the MLE method in^23^, but in these cases, the relation to the *c* value is lost, and thus not accessible for constructing priors, which are multiplicative factors to Eq. (3), to constrain *c*. As described further down, constraints on the estimated fluorophore photon efficiency can be imposed, either statically or dynamically, to exclude spatial regions with very low/high excitation power compared to measured photons as described below. To emphasize the relation between the PSF ratios and the photon ratios, Eq. (3) is in a form with a global exponent, ∑_*i*_*n*_*i*_. The exponent will not change the location of the likelihood peaks and valleys but will make them sharper if increased. This is quantified by the Cramér Rao lower bound (CRLB) for Eq. (3), which will depend on the total number of photons, the PSF shape, and the PSF pattern used. Thus, the underlying principle of the localization by Eq. (3) is to produce a series of photon count ratios {*n*_*i*_/ ∑ *n*_*i*_} for a given excitation pattern and find the position with corresponding mean photon ratios {*λ*_*i*_/ ∑ *λ*_*i*_} which is given by the ratios between PSFs. Background bias can be incorporated within the PSF model; both constant and spatially distributed bias can be added and considered a part of the PSFs. For further discussion and 1D examples, see Supplementary Note 1.

By itself, Eq. (3) needs to be confined to a relatively small region or used in a high photon count setting to produce reliable and correct position estimations. But, experiments are normally photon count-limited and in this regime, the likelihood might have sudden unphysically large peaks that arise due to shot noise and background noise. To counterbalance this problem, we introduce a regularization schema based on weak priors which are multiplied with Eq. (3).

First, a simple constraint on permitted *c* values is introduced by the binary map ${T}_{c}({{{{{{{\bf{r}}}}}}}})=\{1\;{{{{{{{\rm{if}}}}}}}}\,{c}_{min} \, < \, \tilde{c}({{{{{{{\bf{r}}}}}}}}) \, < \, {c}_{max}\;{{{{{{{\rm{else}}}}}}}} \, 0\}$ where *c*_min_ and *c*_max_ are selected depending on the PSF pattern, the PSF shape, and the sample. The upper value is the most important since it excludes regions where the sum of all PSFs is low and close to the noise floor; in these regions, the PSFs has not enough power to excite a fluorophore to obtain the registered photon counts. The second prior targets the interest of keeping the time evolution of estimated *c* values smooth, on average. Over a longer time span, it is assumed that the fluorophore maintains a rather stable *c* value until it bleaches. For that purpose, we assume that we can, over longer time periods, average over the fluorophore blinking time, and use a Gaussian with a fixed width and an exponential weighted moving average as its mean. This approach restricts the possible *c* values dynamically over time. Parameters for the first and second prior are selected such that their effects are very weak. The third and last prior penalizes large jumps within the tracking volume. This is done by applying a Gaussian prior distribution with a fixed width around an exponentially decaying mean over previously estimated positions, see Eqs. (12) and (13). It will suppress large jumps, but also puts an upper limit on the observed diffusion rate. For example, we use a full-width half maximum of 470*nm* at the 0.84*ms* full pattern update rate, which corresponds to a maximal diffusion rate of 24 μm^2^s^−1^. Further discussions and details on the priors and the post-processing steps can be found in the Methods Position estimation section. The final position estimation is obtained by minimization of the negative log-likelihood, Eq. (8). A closed form can be found for simple analytical PSFs and patterns, but for more complicated PSFs and large patterns, a numerical minimization is necessary. In this study, we rely on the latter.

### Implementation

**Fig. 1:**
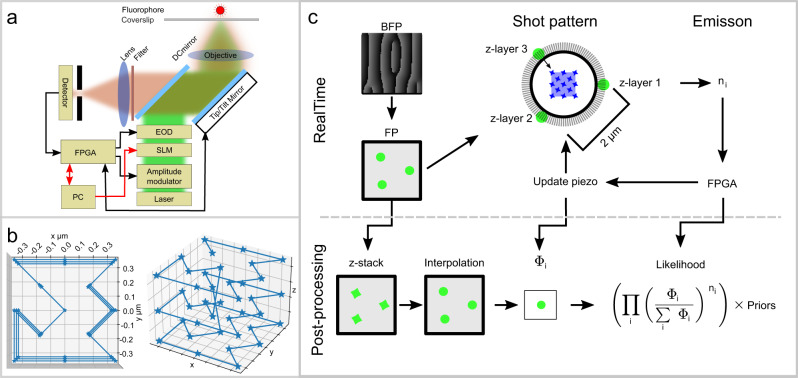
Optical setup and tracking concept. **a** Illustration of the optical setup, laser excitation path with amplitude modulation, spatial light modulator (SLM) for PSF engineering, and electro-optic deflectors (EOD). Following the excitation light path after the dichroic mirror, a piezo-driven tip/tilt mirror scanner is used for long-range scanning, the light path ends with an objective and sample. Collected fluorescence is de-scanned by the tip/tilt mirror and passed through the dichroic mirror, fluorescent filters, and ends with a confocal detection based on single photon counting avalanche photodiodes. **b** Tracking pattern created by the EODʼs . On the left is a view of the pattern from the top, and on the right viewed from the side. Stars indicate the placement of the Gaussian focal points. **c** Graphical representation of the real-time and data post-processing. Real-time; The PSF is shaped at the back focal plane (BFP) so that the desired PSF appears at the focal plane (FP). The center part of the PSF is selected by a 2 μm circular aperture, the tracking pattern is created by moving, using the EODʼs, desired part of the PSF into the center area where the reporter is exposed, simultaneously the aperture blocks the remaining part of the PSF. Emitted photons are detected and processed by the field-programmable gate array (FPGA) to obtain a new piezo position. Post-processing (left to right); the PSF is sampled as a z-stack which is interpolated, and processed with the EOD pattern and piezo center. Along with the photon counts, this fully reconstructs the illumination pattern and gives the parts necessary for constructing the likelihood.

An overview of the optical implementation is shown in Fig. 1a. The excitation laser is passed through an amplitude modulator and a spatial light modulator (SLM) for PSF engineering. Two *xy* scanning systems are used. The first is a fast, short-range *xy* electro-optic deflection (EOD) system, and the second is a piezo-driven tip/tilt mirror scanning system which is placed after the dichroic mirror and used for long-range *xy* scanning or tracking. After both scanning systems, the excitation path ends with an objective that focuses down to the sample which is on an *xyz*-piezo stage. Detection of fluorescence is done by a standard confocal configuration with a large 200 μm pinhole and an SPC-APD. A detailed description of the optical layout can be found in the Methods Position estimation section. For tracking in the axial direction, the z-piezo of the *xyz*-piezo stage is used for slow long-range adaptation. For fast short-range movements, an SLM hologram is programmed to produce a single PSF that consists of three Gaussians. Axially, one of the Gaussians is placed on the focal plane while the other two are shifted 400 nm out of focus in each direction. In the *xy*-plane, the Gaussians are spaced on the perimeter of a circle with a 2 μm diameter. An aperture is placed after the EODs at an intermediate image plane. This aperture selects only the center part of the circle. When activating the EODs, one of the three Gaussians is moved into the circle center to produce the excitation pattern in one z-layer while the other two Gaussian are blocked by the aperture. Switching to a new axial layer is done by removing the Gaussian from the circle center and replacing it with one of the other. During tracking, a 50 kHz pulse schema is used for excitation. The first 10 μs, while the laser is off, is used for moving the beam into place within the aperture and the remaining time is used for excitation. When shifting between z-layers, an extra 10 μs is used for beam movement by blanking out one laser shot. The pattern used is a 13-point *xy* pattern repeated on each of the three z-planes plus 1 blank shot for each move between *z*-layers. The reconstruction volume spans a volume of 950 × 950 × 1390 nm^3^. A representation is found in Fig. 1b where each star indicates Gaussian center positions and the interconnections represent the order of the shots. Other patterns have been suggested, like the knight’s tour^24^ for continuous beam scanning, but for our application, the rationale is the following; the shots in a quadrant in the xy plane spanning the three *z*-planes can by themselves be used for tracking reporters moving relatively slowly, and duplicating this unit covers more volume which enables us to keep the faster-moving reporters that are escaping the center unit. Also, pattern scanning time is optimized by minimizing the number of axial moves, which is done by completing one axial layer before moving to the next. The restriction of 3 Gaussians is only chosen for simplicity to illustrate the principle and to keep the time resolution high while covering a large volume. There is no direct limitation by adding one or two more Gaussians which will create 1 or 2 more *z*-layers. This will have a negative impact on the time resolution but increase the overall spatial resolution (mostly in the axial direction). The pattern used is an empirical optimization where the inner cube without the center shot is enough for slow reporters and is a Gaussian MINFLUX configuration. Adding a second cube outside of this with the same beam spacing expands the volume to accommodate fast reporters.

Emission from each laser shot is recorded by the SPC-APDs and processed by a field-programmable gate array (FPGA). The FPGA is used to raster scan the sample, find tracking reporters, produce the tracking pattern, update the tip/tilt-piezo mirror position by calculating a centroid, as well as bundle all necessary data to be sent to the computer.

For a pattern of Gaussians, a centroid calculation is an optimal position estimator and this is implemented in the FPGA for piezo repositioning. The centroid calculation is an efficient and simple method, but by itself, it does not give an accurate position estimation since the true PSF is not a true Gaussian. Instead, the trajectory estimation is recalculated in a post-processing step by minimizing the cross-entropy together with the priors, see Eq. (8), to estimate **r**_*p*_. To do this, we measure the true excitation PSF, Φ_*i*_, by acquiring an oversampled image z-stack using the EMCCD camera. The image z-stack has voxels of the dimension 80 × 80 × 50 nm^3^ and to avoid quantization error due to voxel size, we interpolated the z-stack to obtain 10 × 10 × 10 nm^3^ voxels; details of how we sample the z-satck and interpolate are described in the Methods PSF engineering section. Together with the EOD pattern, the tip/tilt-piezo position, and the photon counts {*n*_*i*_}, we reproduce the sequence of PSFs {Φ(**r** − **r**_*i*_)} used for tracking and then calculate our position estimation by Eq. (8).

### Experimental evaluation in combination with simulations

**Fig. 2:**
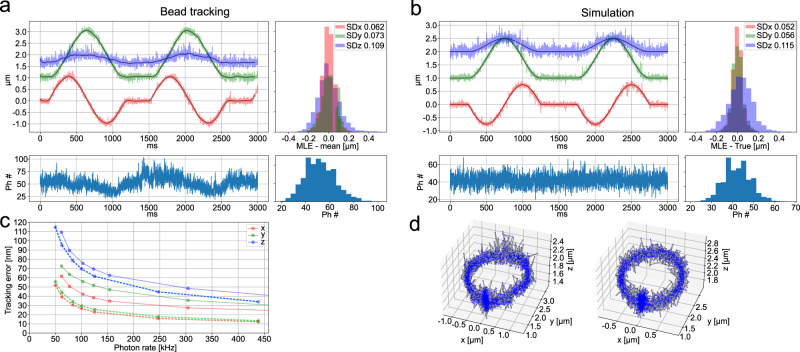
Resolution test and comparison to simulation. **a** Tracking of beads fixed in agarose, the piezo stage is programmed to trace circles with an *x*, *y*-radius of 1 μm and a z peak-to-peak oscillation of 0.5 μm. Upper left; Trajectory estimated *x* (red), *y* (green) and *z* (blue) coordinates with a temporal resolution of 0.84 ms. Upper right; distribution of SD over the trajectory between estimation and a moving average with a window size of 100 points. Bottom left; photon counts per localization which corresponds to 60 kHz photon count rate. To the right is the photon count distribution. **b** Similar to **a** but for a simulated trajectory with an *x*,*y*-radius of 0.75 μm and a z peak-to-peak oscillation of 0.5 μm, the error distribution (upper right) is here the distance to ground truth. **c** Resolution as a function of photon count rate for *x* (red), *y* (green), and z (blue), solid lines are for the bead data, and dashed lines correspond to the simulations. Each point is the SD of the distance histogram in **a** and **b**. These, are obtained by increasing the exponential moving average of the likelihood. **d** 3D point scatter plot of the estimated (left) and simulated (right) trajectory. Source data are provided as a Source Data file.

Evaluation of the real-time tracking system and post-processing is accomplished by tracking immobilized 20 nm beads moved with a piezo stage. The beads are immobilized in agarose and mounted on the sample stage. The stage is programmed to move in a circle with a radius of 1 μm, and perform a sinusoidal pattern with a peak-to-peak amplitude of 0.5 μm in the axial direction. Both movements have a duration of 1 s with a subsequent 0.5*s* pause before the motion is repeated. The bead tracking result is compared to simulated tracking of a fictive fluorescent particle traveling in a path similar to that of the immobilized beads, where the simulated photon counts are generated by the experimentally acquired PSF. These photon counts and PSF positions are then run through the same post-processing as the bead data. In typical experiments, the photon rate is around 60 kHz when the fluorophore is excited with an average power of 24 μW per PSF at the back focal plane of the objective. At this count rate, the spatial resolution is on average 67 nm in the lateral and 109 nm in the axial direction with a time resolution of 0.84ms (Fig. 2a, b). To see how the resolution scales with the photon count rate, we apply an exponential moving average over the likelihood (Eq. (3)) during the reconstruction, see “Methods” Likelihood exponential averaging section. When comparing the bead tracking with the simulations (Fig. 2c), we find that the tracking error is larger for the beads compared to the simulation, although the overall trend is preserved. To further investigate the resolution, we calculated the CRB, see Supplementary Note 6. This suggests that the pattern used is producing a smooth and flat resolution limit in the interior of the tracking volume. In general, the piezo stage appears to follow the prescribed path; however, closer inspection shows clear oscillations in the reconstructed path and that the stage overshoots its mark at the start and end of the pause. We attribute this behavior to the stage’s underdamped feedback loop as well as vibrations induced by the stage motion. These behaviors negatively affect the resolution estimation and imply that the true resolution is likely closer to the theoretical limit of 41 nm lateral and 95 nm axial observed in the simulation. During actual single-molecule tracking, the stage is stationary in the lateral direction and only moves very slowly in the axial direction. To further mitigate any possible stage problems, we turn off the stage piezo feedback system and monitor *z*-axis displacements with a 980 nm laser which is totally internally reflected at the glass water interface during measurements. This approach reduces any stage-induced movements and means that our experimental resolution is better than what can be obtained by tracking a bead moved by the piezo stage. For further implementation details and validation tests, see Supplementary Note 6.

### Simulation in E. coli geometry

**Fig. 3:**
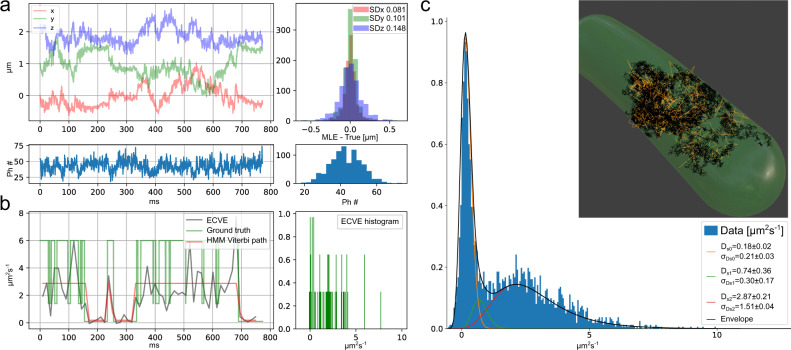
Tracking simulation in an *E. coli*-like geometry. Example trajectory with HMM analysis of 100 simulated trajectories. **a** Upper left; example trajectory with *x* (red) *y* (green) *z* (blue) estimated position coordinates with corresponding photon counts (bottom). Upper right; the deviation away from ground truth with the SD of the distribution in the legend, (bottom) is the photon count distribution. **b** Left; ECVE diffusion point estimation (gray) along the trajectory in (**a**), ground truth diffusion is in green, and the HMM prediction in red. Right; the distribution of estimated diffusions over the trajectory in (**a**). **c** Distribution of all ECVE values from 100 trajectories. The 3 state HMM model prediction of the emission distributions is plotted on top of the histogram, and the inverse-gamma mean and SD of each emission distribution is reported with standard error from a bootstrap analysis over trajectories in the legend. Inset, 3D representation of the cell volume with ground truth trajectory in black and the estimated path in yellow. Source data are provided as a Source Data file.

To get an estimation of the system’s ability to track molecules within cells, we make a diffusion simulation in a replicated E. coli geometry. We use a three-state model with a fast diffusion state (6 μm^2^s^−1^) corresponding to a free protein in the cytoplasm, a slow diffusion state (0.1 μm^2^s^−1^) corresponding to long-lived binding to a larger complex, and a short-lived state with intermediate diffusion (1.4 μm^2^s^−1^) representing interrogations of possible binding sites. See Supplementary Note 5 for the model parameters. The simulation generates 100 trajectories of random lengths between 50 to 800 ms and incorporates motion blur by updating the particle position for each new laser shot in the tracking pattern. In Fig. 3a, a trajectory is shown with its photon counts and distance to the ground truth. The 3D trajectory is seen in Fig. 3c inset where black is the ground truth and the yellow line is a track with 0.84 ms time resolution. The error is larger compared to the bead tracking in Fig. 2 for the same photon count, which is mostly due to motion blur in the fast-moving parts of the trajectory. In the slow-moving parts of the trajectory, the position estimation is more accurate.

### Evaluation of diffusion rates

To analyze each trajectory, a point estimator for diffusion rate is derived for short intervals over the trajectory. We build on the covariance estimator (CVE) approach derived by Berglund et al. and related works^25–27^, and expand their work by incorporating an arbitrary time lag (*j*) within the theory and deriving alternative point estimators. The reasoning here is that in a single step, a trajectory with high temporal resolution at low photon counts is plagued by positioning noise and will benefit from a larger time step to let the diffusion step grow while being less influenced by the localization error. By moving up in the mean square displacement (MSD) curve, changes are accumulated and the positioning error is less dominant. The mean square displacement over an estimated trajectory {*X*_*k*_} is given by the mean over the square of steps Δ_*k*,*j*_ = *X*_*k*+*j*_ − *X*_*k*_ and was shown by Berglund^25^ to be of the form ${\left\langle {{{\Delta }}}_{k,j}^{2}\right\rangle }_{k}=2D(j-2R){{\Delta }}t+2{\sigma }^{2}$ when including motion blur and localization error. Here ${\left\langle \right\rangle }_{k}$ denotes the mean over the parameter *k*, *D* is the diffusion constant, *j* is the number of steps, *R* is a blur factor, Δ*t* is the time of a single step, and *σ* is the positioning error. They continue and show that the covariance matrix between single steps, Δ_*k*,1_, has non-zero elements in the first off-diagonals. They use this relation to derive a point estimator for diffusion. In their treatment, the first step in the mean square displacement is given by the diagonal elements, and by taking the mean over the diagonal elements, they get the first point in the mean square displacement curve. Here we generalize their result further and obtain the covariance matrix for any time lag *j* and covariance between the steps Δ_*k*,*j*_. For derivation details, see Supplementary Note 2. After some calculations, the generalized covariance matrix is given by

4 $$\left\langle {{{\Delta }}}_{k,j}{{{\Delta }}}_{{k}^{{\prime} },j}\right\rangle=\left\{\begin{array}{cc}2D(j-2R){{\Delta }}t+2{\sigma }^{2},&\left|k-{k}^{{\prime} }\right |=0\\ 2D(j-\left|{k}^{{\prime} }-k\right|){{\Delta }}t,&\left|k-{k}^{{\prime} }\right|\, < \, j\\ 2DR{{\Delta }}t-{\sigma }^{2},&\left|k-{k}^{{\prime} }\right |=j\\ 0,&\left|k-{k}^{{\prime} }\right|\, > \, j\end{array}\right.$$

where *j* > 0 and

5 $$R=\frac{1}{{{\Delta }}t}\int\nolimits_{0}^{{{\Delta }}t}S(t)\left(1-S(t)\right)dt$$

which is the blur factor with $S(t)=\int\nolimits_{0}^{t}s({t}^{{\prime} })d{t}^{{\prime} }$ with $s({t}^{{\prime} })$ as the shutter function. The covariance matrix, Eq. (4), tells us that moving along the mean square displacement curve (increasing *j*) indeed generates more non-zero off-diagonal elements, from Berglund’s single step (*j* = 1) with a single off-diagonal to a band of width 2*j* for a step size of *j*. It is worth noting that the localization error (*σ*) is not present for the terms in between the main diagonal and the last. Deriving a simple estimator from this covariance matrix can be done in several ways. The CVE from Berglund et al. is obtained by solving for *D* and *σ*^2^ when *j* = 1. Alternatively, but more complicated and computationally heavy, one can construct a maximum likelihood estimator similar to Shuang et al.^27^ but spanning more than one *j* step. See Supplementary Note 3 for a discussion on this topic. However, for our analysis, we note that for *j* > 1, the off-diagonals between $0 \, < \,|k-{k}^{{\prime} }|\, < \, j$ are all independent of *σ*, and a simple estimator for diffusion would be to solve for *D* and *σ*^2^ for a fixed value of $|k-{k}^{{\prime} }|$. For the first off-diagonal, $|k-{k}^{{\prime} } |=1$, and a time lag of *j*, the estimator is

6 $$\tilde{{D}_{j}}=\frac{{\left\langle {{{\Delta }}}_{k,j}{{{\Delta }}}_{{k}^{{\prime} },j}\right\rangle }_{1}}{2{{\Delta }}t\cdot (j-1)}$$

where ${\left\langle \right\rangle }_{\left|{k}^{{\prime} }-k\right|}$is the mean along the $\left|{k}^{{\prime} }-k\right |=1$ off-diagonal of the covariance matrix, Eq. (4). We will here refer to this estimator as the extended covariance estimator (ECVE). The localization error, *σ*, can be estimated by inserting the diffusion estimation $\tilde{{D}_{j}}$ into the main diagonal, $\left|{k}^{{\prime} }-k\right |=0$, and solving for *σ*^2^. The ECVE estimator is compared to the CVE and the MSD in Supplementary Note 2. The conclusion is that the ECVE is more robust in presence of noise and has less bias compared to the MSD when evaluated over short sections of a trajectory.

### Applying diffusion estimator to simulated data

For diffusion analysis of simulated trajectories, we use the ECVE with a time lag of *j* = 8 and averaging over 16 points. The distribution of all ECVE values is shown in Fig. 3c. By the shape of the histogram, it is clear that it comprises at least two distributions. The first distribution seems to fit well with the slow state, while the second distribution has a peak situated between the intermediate and the fast state. This is expected since the short-lived intermediate state cannot be resolved by the time steps used in the ECVE; a mixture between the intermediate and the fast state is anticipated. In the gray example trajectory shown in Fig. 3b, a clear transition between a slow and a faster state can be seen. The ground truth trajectory (green) confirms the inability to resolve the intermediate and fast states. It is also evident that the ECVE for the higher-diffusion parts of the trajectory is between the fast and the intermediate state. Using all 100 trajectories of the *E. coli* cell simulation, we train a 3-state HMM assuming inverse-gamma emission distributions. In Fig. 3b, the red trace is the predicted Viterbi path, and in Fig. 3c, we show the distribution of all estimated diffusion values together with the predicted inverse-gamma distributions from the trained HMM. However, it is clear that the predicted Viterbi paths do not reflect the dynamics of the true interplay between the intermediate and fast states of the HMM model. Two major distributions can be identified in the histogram. The slow distribution has a mean diffusion of 0.18 ± 0.02 μm^2^s^−1^ (ground truth 0.1 μm^2^s^−1^), with the standard error obtain from bootstrapping over the set of trajectories (see Supplementary Note 4) and 48 ± 9% occupancy (ground truth 43%). The fast state has a mean diffusion of 2.87 ± 0.21 μm^2^s^−1^ (ground truth 6 μm^2^s^−1^) and 45 ± 6% occupancy (ground truth 39%). A third state can be identified between the two main distributions with a low, 7 ± 9% (ground truth 18%), occupancy and a mean diffusion of 0.74 ± 0.36 μm^2^s^−1^ (ground truth 1.4 μm^2^s^−1^). The short dwell time of the intermediate state is the main reason for the ECVE and HMM’s difficulties to find accurate model parameters. In Supplementary Note 5, we prolong the dwell time of the intermediate state and show that a more accurate HMM model fit is obtained.

### Measurements on *E. coli* trigger factor

**Fig. 4:**
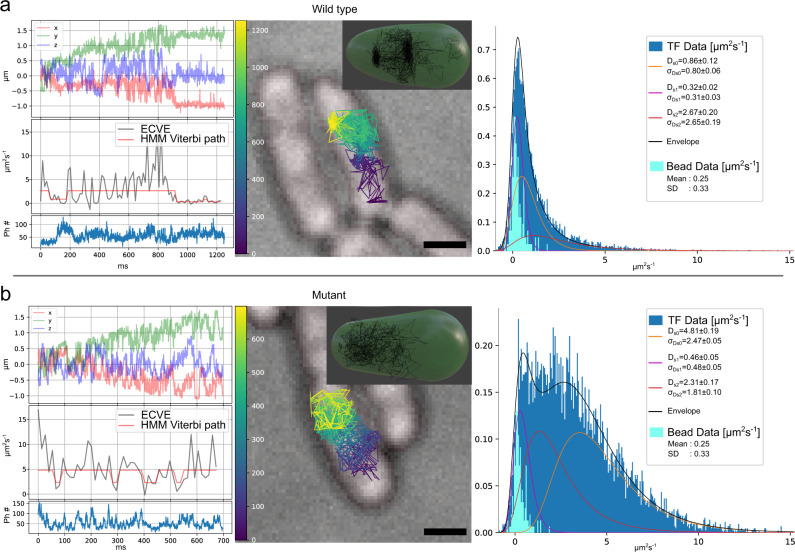
Tracking WT and mutant TF in *E. coli* cells. After the microscope calibration, we conducted two experiments on the same day: one experiment for the wild-type TF where 432 trajectories were acquired from 174 cells, and a second experiment for the mutant TF, where 721 trajectories were acquired from 177 cells. For each sample type, wild-type **a** and mutant **b**, an example trajectory (left) are shown together with the distribution of the accumulated diffusion histograms over all measured trajectories of each type (right side). For each horizontal, the center image is the widefield cell image (black bar 1 μm) with estimated trajectory color-coded over time, and the inset is the 3D trajectory with a cell membrane representation based on the widefield image. The left plots, for the same trajectory shown in the center image, are from the top; position estimation, point-wise diffusion estimation (gray) with HMM Viterbi path (red), and photon counts in the bottom plot. Right plot; in dark blue is the histograms over all diffusion estimates acquired for the WT or Mutant together with the HMM emission distributions. The light blue overlaid histogram is the diffusion estimated distribution for stationary beads with the nanoMax stage in the closed loop configuration, which indicates the lower detection limit at this photon count rate, see also Supplementary Fig. 11. In the histogram, the legends are the mean diffusion values and SD for each HMM estimated distribution with standard error from bootstrapping, as well as the mean and SD of the light blue bead data distribution. Source data are provided as a Source Data file.

The methods developed above are applied to live-cell single-molecule intracellular tracking of the *E. coli* Trigger Factor (TF) chaperone system. One of TF’s functions is to bind to translating ribosomes close to the peptide exit tunnel where it is believed to prevent misfolding of nascent chains during ongoing protein synthesis^28^. Previous studies have captured the binding dynamics of TF and found a rapidly diffusing free state and a ribosome-associated state with slower diffusion due to the large effective size^29^. TF binds to the ribosome surface via its N-terminus. Hence, in order to track TFs in vivo, a reporter protein (HaloTag) was genetically fused to the C-terminus of TF, creating a TF-HaloTag fusion. HaloTag covalently binds fluorescent ligands, such as the organic Janelia Fluor dyes^30^, via a chloroalkane linker^31^. As a control, we constructed a mutant TF in which residues 44–46 (FRK) are exchanged to AAA. These mutations have previously been shown to abolish ribosome binding^32^, and we also observe that the wild-type TF-HaloTag fusion can compensate for deletion of endogenous TF, whereas the mutant TF-HaloTag fusion cannot (Supplementary Fig. 19). For details on the fused HaloTag and mutant construction, see the “Methods” Strain construction and sample preparation section and Supplementary Note 10. Tracking experiments were performed with both the wild-type and the mutant. See Supplementary Note 10 for measurement routines. Example trajectories are shown in Fig. 4. These trajectories are selected to show the dynamical behavior of the WT and the mutant TF, respectively and for that reason, they are longer than the average trajectory. All trajectories were fitted to a 3-state HMM (see Fig. 4a, right side) where the identified HMM is plotted together with the diffusion histogram. For the wild-type (see Fig. 4a), we frequently observe two states at low diffusion, which are interrupted by occasional periods of high diffusion. The occupancies for the slowest (0.32 ± 0.02 μm^2^s^−1^) and the intermediate (0.86 ± 0.12 μm^2^s^−1^) states are 35 ± 6 and 44 ± 7%, respectively and constitute the majority of the events. Only 21 ± 3% of the time, TF is in a faster state (2.67 ± 0.20 μm^2^s^−1^) exploring a larger volume. Considering that elongating ribosomes and free ribosomal subunits have been found to diffuse at 0.03–0.6 μm^2^s^−1^^33–35^, we hypothesize that the two slower diffusion states found by the HMM represent TF bound to ribosomes and/or ribosomal subunits, whereas the fastest state represents freely diffusing TF. The diffusion rates of the two slowest TF diffusion states is high compared to results from camera-based ribosome tracking. This discrepancy can probably be explained by the trade-off between being responsive to rapid movements and at the same time tracking very slow objects at a relativly low photon count rate. To give an estimate of this effect, we track a stationary bead at a photon count rate of 50–60 kHz and a tracking time resolution of 0.84 ms. The diffusion histogram of this stationary bead is scaled and superimposed on the TF diffusion histogram. As seen in Fig. 4 (bead data), the bead distribution has a mean of 0.25 μm^2^s^−1^ and a standard deviation (SD) of 0.33 μm^2^s^−1^ which indicate that the slow TF-diffusion states found by the HMM are likely overestimated. For the mutant TF, which has a lower affinity to ribosomes, HMM fitting of the diffusion trajectories suggests a single slow state (0.46 ± 0.05 μm^2^s^−1^) with low occupancy (14 ± 3%) and two fast states with much higher diffusion than would be expected for ribosome-bound molecules (35 ± 5% at 2.31 ± 0.17 μm^2^s^−1^ and 51 ± 5% at 4.81 ± 0.19 μm^2^s^−1^, respectively). Both the bead and mutant controls reinforce the assignment of the two slower diffusion states in the WT tracking as ribosome binding events. To verify that the whole experiment is reproducible, a second measurement has been conducted. The data, presented in Supplementary Note 7, shows that the results presented here are reproducible.

As a photon flux optimization effort, we also measured the TF and TF mutant with a tracking pattern where the center shot is removed, leaving a hole in the pattern center. The working premise here is that by increasing the distance between Gaussians, the center part of the pattern would be configured for higher resolution at a fixed photon flux. The overall diffusivity results are similar to what is obtained with the full pattern, see Supplementary Note 8. We also noted an increase in the trajectory length for the wild type, see Supplementary Note 9, since the system manages to bring down the photon flux by placing slow-moving reporters at the center where the light intensity is low. No obvious resolution enhancement is observed, but this might be convoluted with the decrease in photon flux at the center.

## Discussion

Single-molecule 3D tracking at the temporal and spatial scale where intracellular biochemistry occurs is highly sought after. Traditional camera based 2D imaging have limitations such as; frame rate, molecules moves out of focus, and limited possibilities to optimize the photon information content. Real-time tracking systems mitigate these limitations, but sacrifice tracking throughput by its limited possibility in simultaneous tracking of molecules. We have here presented a real-time 3D-tracking system capable of tracking fluorescent reporters diffusing up to 10 μm^2^s^−1^ within E. coli bacteria. Fast 3D tracking is enabled by mitigating the workload of inertia-carrying components, like piezo-driven mirrors and stages, resulting in less physical movement when adapting to the fluorescent reporter position. There are two obvious motivations for this. Firstly, mechanical systems are slow and might induce vibrations and noise in the measurements that are difficult to separate from the tracking signal. Secondly, when tracking a diffusing molecule, the randomness in motion is not well suited for a mechanical system which carries inertia. Using a large tracking pattern helps reduce the strain in the mechanical systems and faster molecules can be tracked. Our implementation is based on a single large excitation PSF pattern and cross-entropy-based reconstruction to estimate the position of the reporter over a 950 × 950 × 1390 nm^3^ volume, achieving a spatial resolution of at least 62 × 73 × 109 nm^3^ at a photon count rate of 60*kHz*. For the short-range z-axis beam positioning, we adopt a method which removes the need for a TAG lens or similar for fast but short-range z-scanning. Instead, we construct a single PSF consisting of three Gaussians at different *x*, *y*, *z* locations. With only 2D movement from the EODs, the PSF area corresponding to different *z*-layers is brought into the tracking area where a fluorescent reporter is exposed. For long-range axial coverage, the piezo stage is adaptively moving to keep the reporter close to the focal plane where the photon collection efficiency is highest. Our system is adapted for bacterial cells with a narrow axial profile, but extending to mammalian cells is possible by optimizing the piezo stage for accurate and faster active refocusing. The current speed limit of the system is mostly the update rate of the pattern (0.84 ms for 42 shots). There is a general trade-off between the time-spatial resolution and the volume coverage necessary to keep the reporter within the tracking volume. Currently, systems tracking fast reporters tend to adopt larger patterns which lowers the temporal and spatial resolution. Systems tracking slow reporters can have smaller, spatial-resolution optimized tracking volumes which allow for a high positioning-estimation rate. To some extent, the speed limit of the system is also dependent on the specific PSF used. Our choice of a Gaussian PSF is mostly due to convenience as Gaussians are easy to create and the simple photon count centroid estimation provides a position estimation that can be efficiently implemented within an FPGA for piezo updates. The drawback is that the EOD system must move each Gaussian a long distance, leaving much of the volume uninterrogated for long periods of time. In our case, more than half of the time is spent moving the beam without interrogating the reporter. Pulsed interleaved systems may circumvent this issue but may be difficult to achieve for larger patterns. Our method together with a more optimized PSF offers an alternative. Moving away from Gaussian beams and instead using a large resolution- and speed-optimized static pattern, which has a break in translational symmetry, offers the possibility to shorten the time needed for beam positioning. A first attempt at pattern optimization was made by removing the center shot of the pattern. Although the resolution test (Supplementary Fig. 14) shows a closer agreement with the simulation, this simulation does not show any obvious benefits in resolution compared to the simulation with the full pattern (Fig. 2). What was observed, when tracking the TF with this reduced pattern, was longer trajectories for the wild-type, since the system manages to park the low excitation intensity center over a stationary reporter.

The cross-entropy principle has here been presented for the special case of positioning, but Eq. (3) is not limited to PSFs and positioning. The principle is equally applicable to polarization, wavelength, or other properties where the detected photons obey Poisson statistics and can be related to a known emission/transmission profile that can be altered in a deterministic way.

Extracting valuable information from the tracking data is of equal importance as trajectory building. Molecular dynamics at the single-molecule level can be detected by several approaches, including; multi-color tracking^36^, Fluorescence correlation spectroscopy (FCS)^37^, Single-molecule Förster Resonance Energy Transfer^38^, and polarization enhanced FCS^13^. Attempting to extract information about the binding state by measuring the polarization of the light emitted from the reporter fluorophore was conducted during our experiments but did not reveal information about the conformational changes in the TF when binding to the ribosome. Still, detecting a correlation in conformational states and polarization signal might be possible by introducing a bifunctionally attached dye and acquiring time-tagged photon data for polarization-FCS analysis of trajectories as in ref. ^13^. Although polarization data might carry information about the binding state of the molecule, we did not pursue this path further. Instead, we focused our analysis on the problem of extracting diffusion rates from positioning data on short time intervals. It was found that the MSD gives a poor estimation for this type of problem. The MSD is better suited for systems with only one diffusion rate and a small localization error. The CVE offers an alternative, but since it looks at single steps, big differences in the diffusion rates are required in combination with small localization errors. We introduced the ECVE which connects the MSD and CVE. It gives us the possibility to move up in the MSD curve, where the difference between diffusion rates is more distinguishable, and use the correlations in the covariance matrix to extract the diffusion rate with relatively few data points. An obvious drawback of analyzing displacements over longer time intervals is that we need to assume that the diffusion states have long enough dwell times; if this assumption is not valid, only an average between the diffusion rates will be seen.

In conclusion, we have reached sub-millisecond 3D tracking of rapidly moving macromolecules in bacteria, but we cannot yet resolve short-lived state transitions at this time scale. However, we do believe that the improvements discussed here have the potential to bridge this gap and reach the desired time and spatial resolution where macromolecular interactions occur.

## Methods

### Optics and hardware implementation details

The scanning/tracking microscope software implementation is custom built and based on Labview and Labview FPGA. The EMCCD camera is operated under MicroManager and the focus-tracking system is controlled with a custom Python script.

A detailed view of the real-time tracking microscope is shown in Supplementary Fig. 20 and a table with the equipment used is found in Supplementary Table 1. The working principle of the microscope is as follows; an amplitude modulator based on a Pockel-cell is used for pulsing the laser, this is followed by a TEM00 mode cleaner implemented by a pinhole. From here the beam is expanded and projected on an SLM where we tailor the PSF of the system, see the Method PSF engineering section. Following the beam, we enter into the first *x*/*y*-scanning stage based on EODs. After exiting the first scanning stage, we clean the polarization and rotate the light by half and quarter wave plates. This brings us to the dichroic mirror, which is directly followed by the second scanning stage, a tip/tilt piezo system. From here we bring the laser light to the objective and the sample piezo stage. Collected fluorescence is de-scanned by the second scanning system and transmitted through the dichroic mirror and focused through a pinhole, light is then brought to a wollaston prism and finally to the SPC-APDs.

For widefield imaging the excitation light is coupled to a multimode fiber and at the fiber exit a rotating diffuser is used before entering the microscope through a flip mirror. An EMCCD camera is used for alignment and viewing samples.

Monitoring the sample axial position is done by a 980 nm laser that is passing through the objective in totally internally reflected configuration, the reflected light is collected by a CMOS camera which is triggered each 7.56 ms by the FPGA during tracking.

The real-time tracking system is implemented on an National Instruments PCIe-7852R FPGA. The timing schematic is shown in Supplementary Fig. 21. Three loops are used; the first loop is the 50 kHz loop that controls the piezo and EOD position, this loop is also used for switching between scanning- and tracking-mode. The second loop, is a photon time tagging loop which is running at 200 MHz. This time-tagging loop controls the laser intensity, delays between laser on/off states and the photon measurement on/off states, sampling the piezo stage position, photon time-tagging, and photon accumulation over the measurement window. The third loop calculates a centroid estimation based on a sliding window over two full tracking patterns. This calculation gives the distance to the tracking pattern center and is used in a PID controller, which produces a compensation signal to keep the reporting particle within the tracking region. The PID signal is sent to the first loop, which will update the piezo position. PID parameters are tuned by hand while tracking beads and monitoring the sampled piezo stage signal to avoid large oscillations. When the system is not tracking, a raster scanning is performed. While scanning, the system searches for a tracking candidate. The switch to tracking mode is triggered by a photon count threshold.

### PSF engineering

We engineer the PSF for the excitation light with a spatial light modulator (SLM). The laser is expanded to fill the SLM where a computer-generated hologram (CGH) is drawn. Using the Weighted Gerchberg Saxton algorithm^39^ in combination with aberration corrections patterns, we create three Gaussians at different axial depths.

The z-stack of the engineered excitation PSF is obtained by a reflecting sample placed on the piezo stage. Further, by replacing the emission filter with an OD1 neutral density filter, one can observe the laser reflection onto the EMCCD camera. To test that the dichroic mirror only attenuates the laser and not alter the PSF we place a 50/50 beam splitter between the dichroic and objective. This allows us to sample the PSF with a camera before passing through the dichroic mirror, and confirms that sampling the excitation light through the dichroic mirror can be done. The z-stack is sampled by moving the piezo stage in steps of 25*nm* through the PSF (50 steps), this gives a z-stack range of 2.5 μm with 50 nm between image planes and the in-plane camera resolution of 80*nm*. When creating the PSF we are diffraction limited by the EPI-illumination of the objective, and thus we can safely assume that the light field will be smooth at this scale. The z-stack voxels of size of 80 × 80 × 50 nm^3^ is larger than desired, and to avoid quantization error the *z*-stack is interpolated to obtain 10 × 10 × 10 nm^3^ voxels and cropped to a volume of suitable size. Interpolation is done in two steps, first in *z* and then in the *xy*-plane.

To interpolate over the *z*-axis, a phase retrieval is done by a double weighted Gerchberg Saxton algorithm^40,41^ which iterates between each pair of adjacent images in the *z*-stack. After phase retrieval the *z*-stack represents a light field stack with phase information for each *z*-stack image. By forward and backward propagation of the light field between two adjacent *z*-planes and taking the linear interpolation average of the two light field intensities one obtains any *z*-plane in between.

After *z*-interpolation each *z*-layer is *xy*-interpolated by the OpenCV python package using the bicubic interpolation over 4 × 4 pixel neighborhood.

### Position estimation

During a measurement run, we obtain a set of photon counts, $\{{n}_{i}^{\;j}\}$, where an element ${n}_{i}^{\;j}$ is indexed by *i* ∈ [1, . . , *m*] corresponding to each PSF Φ(**r** − **r**_*i*_) that builds up the full pattern, and *j* ∈ [0, . . , *M* − 1] which are the amount of full patterns acquired during tracking. One full pattern, see Fig. 1, consists of *m* PSF shots. In our implementation *m* = 42 including the three blank shots which are defined to be zero for both photons counts and PSF, thus these do not contribute to the likelihood. Using the sampled PSF (see “Methods” section PSF engineering) together with the tip/tilt mirror piezo position and the EOD position we reconstruct the experimental measured PSF ${{{\Phi }}}_{i}^{\;j}({{{{{{{\bf{r}}}}}}}})={{\Phi }}({{{{{{{\bf{r}}}}}}}}-{{{{{{{{\bf{r}}}}}}}}}_{i}^{\;j})$ with known ${{{{{{{{\bf{r}}}}}}}}}_{i}^{\;j}$ and discretized **r** over a grid of size 950 × 950 × 1390 nm with 10 nm voxels.

Equation (3) can be restated as the negative log likelihood, and with the indexing convention introduced above, it is transformed to

7 $${l}^{\;j}({\{{n}_{i}^{\;j}\}}_{i=1}^{m}|{\{{{{\Phi }}}_{i}^{\;j}({{{{{{{\bf{r}}}}}}}})\}}_{i=1}^{m})=-\ln \left(C\right)- \mathop{\sum }\limits_{i=1}^{m}{n}_{i}^{\;j}\cdot \ln \left(\frac{{{{\Phi }}}_{i}^{\;j}({{{{{{{\bf{r}}}}}}}})}{{\sum }_{i}{{{\Phi }}}_{i}^{\;j}({{{{{{{\bf{r}}}}}}}})}\right)$$

here the second part can be identified as the cross-entropy. When tracking slow moving reporters, at high signal-to-background ratio, with a well behaving PSF in a small search volume, one can seek a position estimation directly from Eq. (7) by ${\tilde{{{{{{{{\bf{r}}}}}}}}}}_{p}^{\;j}=\arg \min ({l}^{\;j}({\{{n}_{i}^{\;j}\}}_{i=1}^{m}|{\{{{{\Phi }}}_{i}^{\;j}({{{{{{{\bf{r}}}}}}}})\}}_{i=1}^{m}))$ over possible positions **r**. However, it is necessary to regularize Eq. (7) for larger search volumes when the signal-to-background ratio is decreased and/or the tracking reporter is fast moving. One regularization method is to use weak priors. Here, we use time dependent physics motivated priors to regularize possible estimations. We seek an estimator of the form

8 $${\tilde{{{{{{{{\bf{r}}}}}}}}}}_{p}^{\;j}=\arg \mathop{\min }\nolimits_{\bar{r}}\left({l}^{\;j}({\{{n}_{i}^{\;j}\}}_{i=1}^{m}|{\{{{{\Phi }}}_{i}^{\;j}({{{{{{{\bf{r}}}}}}}})\}}_{i=1}^{m})+{T}_{c}^{\;j}({{{{{{{\bf{r}}}}}}}})+{G}_{c}^{\;j}({{{{{{{\bf{r}}}}}}}})+{G}_{r}^{\;j}({{{{{{{\bf{r}}}}}}}})\right)$$

where each of the three priors are defined in the next three paragraphs.

For the first prior, ${T}_{c}^{\;j}({{{{{{{\bf{r}}}}}}}})$, in Eq. (8) we bound the fluorophore photon efficiency (Eq. (2)) to exclude regions that fall outside an expected range. Given a photon measurement sequence the prior will effectively exclude regions in the search volume that has a fluorophore photon efficiency that is too high or too low. This binary map, after taking the logarithm, is given by

9 $${T}_{c}^{\;j}({{{{{{{\bf{r}}}}}}}})=\left\{\begin{array}{l}0\,{{{{{{{\rm{if}}}}}}}}\,{c}_{{{{{{\rm{min}}}}}}} \, < \, \frac{\mathop{\sum }\nolimits_{i=1}^{m}{n}_{i}^{\;j}}{\mathop{\sum }\nolimits_{i=1}^{m}{{{\Phi }}}_{i}^{\;j}({{{{{{{\bf{r}}}}}}}})} \, < \, {c}_{{{{{{\rm{max}}}}}}}\\ \infty \hfill\end{array}\right.$$

where *c*_min_ and *c*_max_ are selected depending on the pattern and PSF shape used. In practice ${T}_{c}^{\;j}({{{{{{{\bf{r}}}}}}}})$ is independent of *j*, but there might be situations where the PSF pattern is either more dynamic and the binary maps are not the same between *j*’s or that bounds need to change depending on the tracking situation. In our experiments, the upper bound is set high enough to not affect the likelihood.

For the second prior, ${G}_{c}^{\;j}({{{{{{{\bf{r}}}}}}}})$ in Eq. (8), a more dynamic but tighter constraint is of interest for the fluorophore photon efficiency. Over a longer time span, it is assumed that the mean photon counts per excitation power is changing rather smoothly. To incorporate this as a weak prior we make an exponentially weighted mean of *c* values obtained from estimated positions ${\tilde{{{{{{{{\bf{r}}}}}}}}}}_{p}^{\;j}$, this is defined in a recursive way by

10 $$\hat{c}({\tilde{{{{{{{{\bf{r}}}}}}}}}}_{p}^{\;j})=\left\{\begin{array}{l}0\,{{{{{{{\rm{for}}}}}}}}\,j=0 \hfill\\ (1-{\gamma }_{c})\cdot \hat{c}({\tilde{{{{{{{{\bf{r}}}}}}}}}}_{p}^{\;j-1})+{\gamma }_{c}\cdot c({\tilde{{{{{{{{\bf{r}}}}}}}}}}_{p}^{\;j-1})\,{{{{{{{\rm{for}}}}}}}}\,j \, > \, 0\end{array}\right.$$

where *γ*_*c*_ is the weight parameter, and is set to have a long tail that will average over blinking events. With this averaging, a gaussian prior is constructed and after taking the logarithm gives

11 $${G}_{c}^{\;j}({{{{{{{\bf{r}}}}}}}})=\left\{\begin{array}{l}0\,{{{{{{{\rm{for}}}}}}}}\,j=0 \hfill\\ \frac{1}{2\cdot {\sigma }_{c}^{2}}{\left(\hat{c}({\tilde{{{{{{{{\bf{r}}}}}}}}}}_{p}^{\;j})-\frac{\mathop{\sum }\nolimits_{i=1}^{m}{n}_{i}^{\;j}}{\mathop{\sum }\nolimits_{i=1}^{m}{{{\Phi }}}_{i}^{\;j}({{{{{{{\bf{r}}}}}}}})}\right)}^{2}\end{array}\right.$$

where *σ*_*c*_ is a fixed parameter defining the allowed span of the gaussian.

The thired and last prior,${G}_{r}^{\;j}({{{{{{{\bf{r}}}}}}}})$ in Eq. (8), is a gaussian prior on the previous position estimation. Its effect is to suppress long jumps, assuming that the next position estimation is probably close to the previous estimated positions. Here we use an exponentially weighted mean on the previous estimated positions

12 $$\hat{{{{{{{{\bf{r}}}}}}}}}({\tilde{{{{{{{{\bf{r}}}}}}}}}}_{p}^{\;j})=\left\{\begin{array}{l}{{{{{{{\rm{center}}}}}}}}\,{{{{{{{\rm{position}}}}}}}}\,{{{{{{{\rm{for}}}}}}}}\,j=0 \hfill\\ (1-{\gamma }_{r})\cdot \hat{{{{{{{{\bf{r}}}}}}}}}({\tilde{{{{{{{{\bf{r}}}}}}}}}}_{p}^{\;j-1})+{\gamma }_{r}\cdot {\tilde{{{{{{{{\bf{r}}}}}}}}}}_{p}^{\;j-1}\,{{{{{{{\rm{for}}}}}}}}\,j \, > \, 0\end{array}\right.$$

where *γ*_*r*_ is the weight parameter, and is here chosen to have a short tail. The prior is then given by

13 $${G}_{r}^{\;j}({{{{{{{\bf{r}}}}}}}})=\left\{\begin{array}{l}0\,{{{{{{{\rm{for}}}}}}}}\,j=0 \hfill\\ \frac{1}{2\cdot {{{{{{{{\boldsymbol{\sigma }}}}}}}}}_{r}^{2}}{\left(\hat{{{{{{{{\bf{r}}}}}}}}}({\tilde{{{{{{{{\bf{r}}}}}}}}}}_{p}^{\;j})-{{{{{{{\bf{r}}}}}}}}\right)}^{2}\end{array}\right.$$

where ***σ***_*r*_ = (*σ*_*x*_, *σ*_*y*_, *σ*_*z*_) is a fixed vector of values defining the reach of the gaussian. The value is set depending on the sampling speed in relation to expected max diffusion. We use a full width half maximum of 470 nm at 0.84 ms full pattern update rate, and this corresponds to an upper limit of 24 μm^2^s^−1^ for the possible diffusion rates.

### Likelihood exponential averaging

Increasing the resolution can be done by increasing photon counts. In post-processing we can sacrifice timing bandwidth assuming that the reporter is moving slowly. A way to indirectly do this is to take the mean of estimated positions to get a refined position. Or one can sum photon counts emanating from several PSF shots at a position and estimate a positions with these photons. Here, we instead construct an exponential averaging of the likelihood before adding any priors. The benefit of doing this in this way is that one is updating the current likelihood by taking into account the history of past likelihoods in a trailing fashion. For this, we construct a likelihood with a tail as

14 $${l}_{{{{{{\rm{tail}}}}}}}^{\;j}({{{{{{{\bf{r}}}}}}}})={r}_{{{{{{\rm{tail}}}}}}}\cdot {l}^{\;j}({\{{n}_{i}^{\;j}\}}_{i=1}^{m}|{\{{{{\Phi }}}_{i}^{\;j}({{{{{{{\bf{r}}}}}}}})\}}_{i=1}^{m})+(1-{r}_{{{{{{\rm{tail}}}}}}})\cdot {l}_{{{{{{\rm{tail}}}}}}}^{\;j-1}$$

where *r*_tail_ is how much tail that should be considered, if *r*_tail_ = 1 then we are only considering current data without any historical considerations. The priors are only added after, thus the final position estimation is

15 $${\tilde{{{{{{{{\bf{r}}}}}}}}}}_{p}^{\;j}=\arg \mathop{\min }\nolimits_{{{{{{{{\bf{r}}}}}}}}}({l}_{{{{{{\rm{tail}}}}}}}^{\;j}({{{{{{{\bf{r}}}}}}}})+{T}_{c}^{\;j}({{{{{{{\bf{r}}}}}}}})+{G}_{c}^{\;j}({{{{{{{\bf{r}}}}}}}})+{G}_{r}^{\;j}({{{{{{{\bf{r}}}}}}}}))$$

This averaging is only applied when testing resolution for beads and simulations, for all TF data we disable this averaging by setting the *r*_tail_ parameter to one.

### Strain construction and sample preparation

Tracking of TF-HaloTag was performed in *Escherichia coli MG*1655Δ*tig::kan* in which *tig*, encoding Trigger Factor, was deleted from the chromosome by *λ* Red assisted recombineering^42^ and replaced by a kanamycin resistance marker. Recombineering was done using primers tig_del_F and tig_del_R (Supplementary Table 2) with pKD4 (Addgene #45605) and K-12 MG1655 genome (accession #U00096) as templates. The strain was supplemented with a pQE30lacIq plasmid encoding a TF-HaloTag fusion under the IPTG-inducible T5 promoter, constructed by Gibson Assembly (New England Biolabs) and primers tig_GA_F, tig_GA_R, pQE_GAtig_F and pQE_GAtig_R (Supplementary Table 2). The TF stop codon was replaced by ggc, creating a C-terminal fusion with one G linker. HaloTag was shown not to interfere with the ribosome binding function of TF (see Supplementary Note 10). The TF-HaloTag FRK/AAA mutant plasmid was created by mutagenesis PCR of the previously described plasmid with primers tig_mut44_46_F and tig_mut44_46_R (Supplementary Table 2). All constructs were verified by Sanger sequencing. Tracking experiments were performed on uninduced cells, with only leakage level of TF, to reduce the fluorophore signal.

Cells from a glycerol stock were inoculated in Luria Broth (LB), 50 μg⋅ml^−1^ kanamycin and 100 μg⋅ml^−1^ carbenicillin and incubated at 37 °C 200 rpm overnight. The overnight culture was diluted 1:100 in 10 ml fresh LB supplemented with antibiotics and grown at 37 ^∘^C with shaking until *OD*_600_ reached ca 0.5. Cells were harvested at 4000 × *g*, washed in 1 ml M9 media supplemented with 0.2% glucose and resuspended in 150 μl EZ Rich Defined Medium (RDM, Teknova) supplemented with 0.2% glucose and 0.1 *μM* Janelia Fluor-549 HaloTag ligand. Cells were labeled for 30 min at 25 ^*∘*^*C*. Excess dye was washed off by adding 1 ml M9 followed by pelleting and resuspension in 1 ml M9. The washing was repeated twice, followed by incubation in 2 ml RDM at 37^∘^*C* with shaking for 60 min to further remove excess dye. After incubation, cells were washed three additional times and resuspended to an *OD*_600_ of 0.03 in RDM. The cell suspension was sparsely spread onto a 2% agarose (SeaPlaque GTG Agarose, Lonza) pad in RDM which had been prepared on a 76 × 26 mm microscopy slide (VWR) with a 1.7 × 32.8 cm Gene Frame (ThermoFisher Scientific) attached and covered with a 24 × 32 mm high precision cover glass (ThorLabs).

### Statistics & reproducibility

All experiments were conducted using the same set-up. All data are acquired from cell samples by the following procedure: first we calibrate the microscope by optimizing the PSF and measuring the PSF z-stack. The bead sample is then mounted and tracking of beads is done to see that the microscope is performing as expected. During the microscope calibration cell samples are prepeard. After calibration the cell sample is mounted, moving through the sample colonies coordinates are registered, and a widefield image is taken. About 20 micro colonies are registered for each sample. Each colony is then re-visited to verify that the cells are growing. At this stage a new widefield image is acquired together with a fluorescent image to verify that fluorescence is present. The sample is scanned before turning on the real-time tracking. All data is saved before moving to the next cell colony. Each experiment constitute of measuring both the WT and the mutant, within the same day and with the same microscope calibration. Three such experiments where conducted; two for the full pattern configuration and one with the patten missing the center laser shot. No statistical method was used to predetermine sample size, the experiments were not randomized and the investigators were not blinded to allocation during experiments and outcome assessment.

Statistics are either presented as SD of obtained distributions, this includes tracking errors and comparison of diffusion estimators. Or, statistics are presented as estimated mean, SD, and occupancy from the HMM model fitting where errors are reported as standard errors obtianed by bootstrapping.

### Reporting summary

Further information on research design is available in the Nature Portfolio Reporting Summary linked to this article.

## Supplementary information

Supplementary information

Reporting Summary

## Data Availability

Raw data together with all analysis objects are available at 10.17044/scilifelab.21602844. Strains are available upon request to J.E. Source data are provided with this paper.

## Source data

Source Data

## Code availability

Code developed for analyses is available at 10.17044/scilifelab.21602844.
