# Supplementary material for: Real-time single-molecule 3D tracking in E. coli based on cross-entropy minimization
Source: Nat Commun. 2023 Mar 11;14:1336. doi: 10.1038/s41467-023-36879-1 (PMC10008558; doi:10.1038/s41467-023-36879-1)
Supplement: Supplementary file 1 — Supplementary information [file 41467_2023_36879_MOESM1_ESM.pdf]

SUPPLEMENTARY INFORMATION

Supplementary information for Real-time single-molecule 3D tracking in E. coli based on cross-entropy minimization

Elias Amselem, Bo Broadwater, Tora Hävermark, Magnus Johansson and Johan Elf

Contents

A: Supplementary Note 1 2
A-A Linear intensity 2
A-B Gaussian 3

B: Supplementary Note 2 5

C: Supplementary Note 3 9

D: Supplementary Note 4 10
D-A Hidden Markov Model for estimated diffusion traces 10
D-B Gamma and inverse-Gamma distribution relation 10
D-C Handling negative values when fitting inverse-gamma distributions 10
D-D Bootstrap sampling for error estimation 11

E: Supplementary Note 5 12

F: Supplementary Note 6 14
F-A Circular motion 14
F-B Cramer-Rao lower bound 14

G: Supplementary Note 7 18

H: Supplementary Note 8 19

I: Supplementary Note 9 23

J: Supplementary Note 10 24
J-A Bead sample 24
J-B Assessment of TF-HaloTag *in vivo* function 24

K: Supplementary Tables 25

L: Supplementary Figures 27

A
Supplementary Note 1

Here we give a few 1D examples that highlight the principles behind localization by cross-entropy minimization. Starting from equation 3 it is worth noting that taking the natural logarithm of the likelihood and negating we get

$$l(\{n_i\}|\{\Phi_i\}) = -\ln(C) - \sum_i n_i \cdot \ln\left(\frac{\Phi_i}{\sum_i \Phi_i}\right) \tag{16}$$

the second part can be identified as the cross-entropy between photon counts n_i and the PSFs $\Phi_i = \Phi(\mathbf{r} - \mathbf{r}_i)$ indexed by i, where $\mathbf{r}$ is $\mathbf{r} = (x, y, z)$ for the 3D case and $\mathbf{r} = x$ for the 1D case. The maximum resolution aloud by the model is quantified by the Cramer-Rao lower bound (CRLB) which can be stated as

$$var(\tilde{x}) \geq \frac{1}{E\left[\frac{\partial^2}{\partial x^2} l(\{n_i\}|\{\Phi_i(x)\})\right]} \tag{17}$$

A. *Linear intensity*

If we consider a small region of the PSFs we can within that region linearize it to

$$\phi_i(x) = k_i \cdot x + m_i \tag{18}$$

for this 1D problem consider two PSFs indexed with $i = 1, 2$. Inserting this into equation 16 and setting the first partial derivative of x to zero we get the unbiased estimator

$$\tilde{x} = \frac{n_1 m_2 - n_2 m_1}{n_2 k_1 - n_1 k_2} \tag{19}$$

This estimator is valid in an interval where both PSFs are positive, the details of the bounds will depend on the exact k_i and m_i values. The bounds will be pairs of values given by $-\frac{m_2}{k_2}$, $-\frac{m_1}{k_1}$ or $\pm\infty$, and are independent of the photon count. Increasing the total count, $n_1 + n_2$, will result in a denser position map since there are more combinations of n_1 and n_2 values. As an example consider a triangular 1D-PSF as shown in Fig. 1. We obtain different estimators depending on which x-axis section we are looking at and how much offset is between the red and blue curve. In Fig. 1**a** there are two regions, R1 and R2, and for each region an estimator is given (see the table in Fig. 1**a**). The only difference between the two regions is the reversal of the sign between photon counts. Note that the estimator range is within $\pm m/k$ which is the range where both PSFs are positive. Also, the scaling indicates that given a fixed number of photons ($n_1 + n_2$) the possible positions will be denser with a smaller m/k ratio. This is the same as saying that the resolution is increasing by making m/k smaller, but this will sacrifice range. In contrast, increasing the total photon counts will instead increase the density of points while keeping the range.

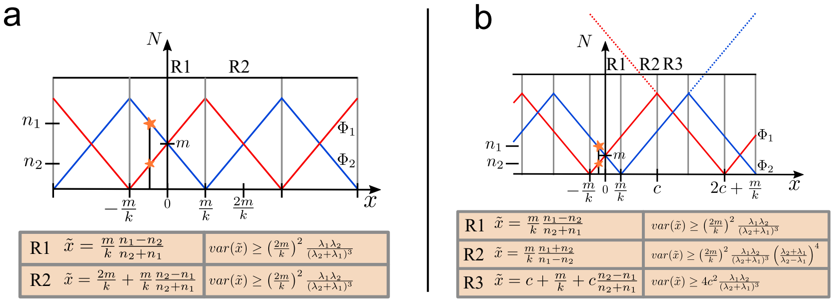

R1	$\tilde{x} = \frac{m}{k}\frac{n_1 - n_2}{n_2 + n_1}$	$var(\tilde{x}) \geq \left(\frac{2m}{k}\right)^2 \frac{\lambda_1\lambda_2}{(\lambda_2+\lambda_1)^3}$
R2	$\tilde{x} = \frac{2m}{k} + \frac{m}{k}\frac{n_2 - n_1}{n_2 + n_1}$	$var(\tilde{x}) \geq \left(\frac{2m}{k}\right)^2 \frac{\lambda_1\lambda_2}{(\lambda_2+\lambda_1)^3}$

R1	$\tilde{x} = \frac{m}{k}\frac{n_1 - n_2}{n_2 + n_1}$	$var(\tilde{x}) \geq \left(\frac{2m}{k}\right)^2 \frac{\lambda_1\lambda_2}{(\lambda_2+\lambda_1)^3}$
R2	$\tilde{x} = \frac{m}{k}\frac{n_1 + n_2}{n_1 - n_2}$	$var(\tilde{x}) \geq \left(\frac{2m}{k}\right)^2 \frac{\lambda_1\lambda_2}{(\lambda_2+\lambda_1)^3}\left(\frac{\lambda_2+\lambda_1}{\lambda_2-\lambda_1}\right)^4$
R3	$\tilde{x} = c + \frac{m}{k} + c\frac{n_2 - n_1}{n_2 + n_1}$	$var(\tilde{x}) \geq 4c^2 \frac{\lambda_1\lambda_2}{(\lambda_2+\lambda_1)^3}$

Fig. 1. **Linear intensity 1D PSF. a**, Plot shows two triangle 1D-PSFs, red and blue, over the x-axis with the y-axis being their respective intensity. A fluorophore at a position on the x-axis will emit photons proportional to each PSF intensity (marked as stars). Regions where estimators are valid are marked with gray vertical lines. In the table under the plot are the estimators for the regions R1 and R2. The first column is the estimator and the second is the CRB. **b**, the same as **a** but the two PSF have a different offset between each other. Three regions are identified, R1, R2 and R3. For each region the estimator is shown in the table under the plot. Note that each estimator is only valid within the interval marked by the gray lines in the plot and are given by the interval $\pm m/k$ for R1, m/k to c for R2 and the R3 estimator is valid within the interval c to $c + 2m/k$. Still the estimator itself is not bounded by the interval. If photon shot noise is introduced when using the R2 region the estimator might give a wrong position since the estimator has a range from m/k to ∞.

The CRB is calculated using equation 17 and is given by

$$var(\tilde{x}) \geq \frac{(k_1 \cdot \tilde{x} + m_1)(k_2 \cdot \tilde{x} + m_2)((k_1 + k_2) \cdot \tilde{x} + m_1 + m_2)}{(k_1 m_2 - k_2 m_1)(\lambda_2 k_1 - \lambda_1 k_2)} = \frac{\lambda_1 \lambda_2 (k_1 m_2 - k_2 m_1)^2 (\lambda_1 + \lambda_2)}{(\lambda_2 k_1 - \lambda_1 k_2)^4}$$

where λ_i are the corresponding mean photon counts. As seen in the table in Fig. 1**a**, the CRB (see right column in table) gives the m/k dependence but does not indicate anything about the range dependence. If another offset is set between the two PSFs, we get other regions and estimators. For the case in Fig. 1**b**, we have three regions. The first region (R1) is as in Fig. 1**a** but with a shorter range since the m value has decreased and is bounded in the interval $\pm m/k$. The second region (R2) has two parallel PSF intensity curves. The estimator gives values from m/k to ∞ which is valid if these continue to increase, but as seen in the plot the valid range is from m/k to c. The last term in the CRB for R2 shows that the resolution drops further away from the point m/k we get. The third region (R3) is as the first but with an intensity offset, this offset increases as c increases. The estimator has the possible range of m/k to $2c + m/k$, but for our example it is only valid within c to $c + 2m/k$. From the CRB for the R3 region we do see that the resolution goes down as c increases.

Thus, to optimize the resolution, given fixed mean photon counts, one can either increase the slope while keeping the intensity bias fixed or have the PSFs intersect close to the low amplitude part and keep the slope fixed. The first option depends on the optical hardware used and has often limited possibilities for improvement. Some optimization can be done by selecting an appropriate PSF and pattern; however for most applications Gaussian or doughnut beams are preferred, and the corresponding optics are configured optimally for highest possible resolution. The second possibility for resolution optimization is the m value, here the limitations are related to background noise, PSF low intensity lobes, and excitation power. Our example in Fig. 1 is not realistic, in reality there are optical bandwidth limitations which couple the PSF m and k values. For example, a zero mean Gaussian beam has an m/k ratio scaling as $1/x$ indicating an increase of resolution by moving the Gaussian PSFs apart from each other. In experiments, true Gaussians are not possible. Instead the PSF has lobes which will limit resolution by separation. For doughnut beams, there are two regions: one close to the center that has a ratio scaling as x and a second region, which is the outer part of the doughnut, that scales as the Gaussian. Thus, for the doughnut center the resolution decreases as the displacement is increased.

B. *Gaussian*

As a second example we describe the special case of a 1D Gaussian PSF. Consider a PSF described by

$$G(x, x_i) = A \cdot e^{-\frac{(x-x_i)^2}{2\sigma^2}} \tag{20}$$

where x_i is the offset, σ is the PSF SD, and A is related to the beam intensity. For this 1D case, we need at least two beams for a localization, $i \in [1, 2]$, and without loss of generality, we can assume that these are placed symmetrically around the origin, $x_{1,2} = \pm\alpha$. Inserting our assumptions into equation 3 gives the likelihood function

$$L(x) = C \cdot \left(\frac{1}{1 + e^{\frac{2\alpha}{\sigma^2}x}}\right)^{n_1} \times \left(\frac{1}{1 + e^{-\frac{2\alpha}{\sigma^2}x}}\right)^{n_2} = C \cdot (g_1(x))^{n_1} \cdot (g_2(x))^{n_2} \tag{21}$$

In Fig.(2) we plot $g_1(x)$ and $g_2(x)$ for different values of $2\alpha/\sigma^2$, as seen the slope increases as $2\alpha/\sigma^2$ increases. This indicates an increased resolution but only at the center, further out the resolution is decreasing.

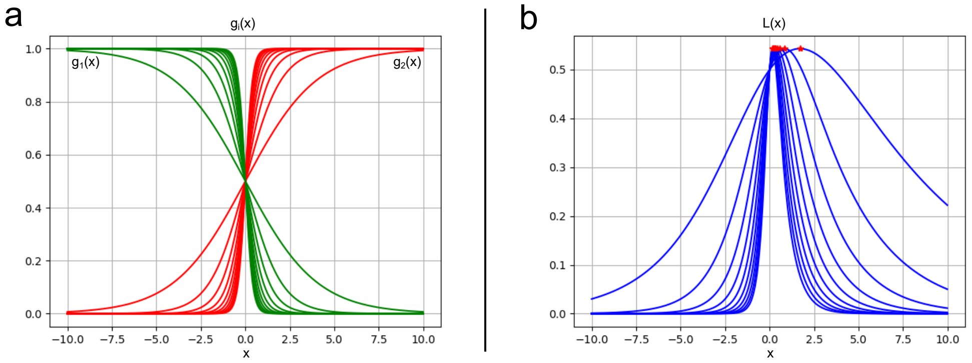

Fig. 2. **1D Gaussian position estimation principle. a**, plot shows curves for the $g_i(x)$ part of equation 21 with $2\alpha/\sigma^2 = k$, $k = [1, .., 10]/2$. As k increases the steeper the function transits from 0 to 1. Red is $g_1(x)$ and green is $g_2(x)$. **b**, plot is $L(x) = g_2^{0.3} \cdot g_1^{0.7}$which represent the likelihood over the positions x for different k values. The exponent corresponds to the percentage of the mean photon counts, λ_i, with $0.3 = \lambda_2/(\lambda_1 + \lambda_2)$ and $0.7 = \lambda_1/(\lambda_1 + \lambda_2)$. As seen the likelihood is more narrow for high k values. The red points indicate the maximum, they do not coincide since for different k values the photon counts used represent different positions.

Assuming that there is a fluorophore at position x_p generating photon counts, we have the problem $L(x_p|\{n_i\})$. For a general PSF together with a given pattern there might be several maxima, thus finding a unique maximum likelihood estimator might not be possible without further assumptions. However, for the Gaussian example, there is a unique solution. Following the standard maximum likelihood procedure gives

$$\hat{x}_p = \frac{\sigma^2}{2\alpha} \ln\left(\frac{n_1}{n_2}\right) = \frac{\sigma^2}{2\alpha} \ln\left(\frac{n_1}{n_1+n_2}\right) - \frac{\sigma^2}{2\alpha} \ln\left(\frac{n_2}{n_1+n_2}\right) \tag{22}$$

which is our unbiased estimator. The localization performance can be calculated from equation 17 and equation 21 which gives

$$var(\hat{x}_p) \geq \frac{\sigma^4}{4\alpha^2} \frac{\left(1+e^{\frac{2x\alpha}{\sigma^2}}\right)\left(e^{-\frac{2x\alpha}{\sigma^2}}+1\right)}{(\lambda_1+\lambda_2)} = \left(\frac{\sigma^2}{2\alpha}\right)^2 \left(\frac{1}{\lambda_2}+\frac{1}{\lambda_1}\right) \tag{23}$$

where λ_i are the mean photon counts. As can be seen the CRB is not uniform over x, as we move further out from the center we gradually lose resolution. As expected the resolution decreases when the mean photon rate decreases. As long as one is at the center, it is more beneficial to increase the distance, 2α, between the beams or decreasing σ compared to increasing the photon count by higher excitation power. This can also be seen from equation 22 where for any non zero, finite total photon number, n_1+n_2, the estimator covers all space, with the limits $\pm\infty$ when n_1 or n_2 are zero. But the total number of possible points between $\pm\infty$ will be finite, more precisely n_1+n_2-1, and these will span an interval on the x axis of length $(\sigma^2/\alpha)\ln(n_1+n_2-1)$. Thus increasing the distance between the beams will result in a higher sampling density at the center. But this effectively decreases the interval when we exclude extreme points at $\pm\infty$. In contrast, increasing the total photon count will add points over the whole interval. Thus, for imaging applications where only slow movements of the reporter are present a resolution optimization using the beam configuration can be done by sacrificing range. But, if the reporter is moving one needs to increase the range of the estimator by sacrificing resolution.

B
SUPPLEMENTARY NOTE 2

Determining Diffusion Coefficients

Given position estimations for a particle trajectory, we like to find relevant parameters that characterize the underlying dynamics related to it. For dynamical processes where the molecule in question is driven by a diffusion process and associates/dissociates to a larger molecule, it is expected that the diffusion state-change can be observed. A more careful treatment of MSD where contributions from motion blur and position estimation noise are incorporated reveals a richer theory with alternative estimators. Building on the covariance point estimator (CVE) approach, that was derived by Berglund et al. and related work [1–3], we expand their work further to incorporate an arbitrary time lag within the theory and derive alternative point estimators.

Suppose that a particle is moving in one dimension by pure Brownian motion with a diffusion coefficient D and that the particle position X_k for $k = 1, ..., N$ is estimated with a time interval of Δt, which will be referred to as the frame time, and k is the frame index. For higher dimensions, 2D and 3D motion, we are assuming that each dimension is independent and each can be treated separately. The observed position of the particle is then the average of its position weighted by a "shutter function" $s(t)$, a non-negative function whose integral over the frame time interval is unity. On top of this, we add a noise factor representing the localization noise term which we assume is a zero mean gaussian with covariance $\langle \epsilon_i \epsilon_j \rangle = \sigma^2 \delta_{i,j}$. Given that we have N frames, the kth frame ending at time $t = k \cdot \Delta t$ where $k = 1, ..., N$, gives an observed position X_k given by

$$X_k = \int_{(k-1)\Delta t}^{k\Delta t} s(t' - (k-1)\Delta t) X_{true}(t') dt' + \epsilon_k \tag{24}$$

where $X_{true}(t')$ is the true position of the particle at time t' and ϵ_k is the value of the additive localization noise in frame k. In the ideal case when $\epsilon_k = 0$ and $s(t)$ is a delta function then the observed position is the true position and the distribution of position intervals $\Delta_{k,j} = X_{k+j} - X_k$ for any fixed $j > 0$ are independent and are zero mean Gaussian distributed with a variance $\left\langle \Delta_{k,j}^2 \right\rangle = 2 \cdot D \cdot \Delta t \cdot j$ and the covariance matrix off-diagonal elements $\langle \Delta_{k,j} \Delta_{k',j} \rangle = 0$ for all $k \neq k'$. Introducing localization noise and blur will induce correlations between $\Delta_{k,j}$ which will give a covariance matrix with nonzero off-diagonal elements. Using equation 24 it is straightforward to show that $\langle \Delta_{k,j} \rangle = 0$. For obtain the covariance we like to evaluate $\langle \Delta_{k,j} \Delta_{k',j} \rangle$, and after some algebra and using the Brownian motion property $\langle X_{true}(t'') X_{true}(t') \rangle = X_{true}(0)^2 + 2D \min(t', t'')$ and that $\min(x, y) = (x + y)/2 - |x - y|/2$ we get the covariance matrix

$$\langle \Delta_{k,j} \Delta_{k',j} \rangle = \begin{cases} 2D(j - 2R)\Delta t + 2\sigma^2, & |k - k'| = 0 \\ 2D(j - |k' - k|)\Delta t, & |k - k'| < j \\ 2DR\Delta t - \sigma^2, & |k - k'| = j \\ 0, & |k - k'| > j \end{cases} \tag{25}$$

where $j > 0$ and

$$\begin{aligned} R = & \tfrac{1}{\Delta t} \Big(\int_0^{\Delta t} s(t') t' \cdot dt' \\ & - \int_0^{\Delta t} \int_0^{\Delta t} s(t') s(t'') \cdot \min(t', t'') dt'' dt' \Big) \\ & = \tfrac{1}{\Delta t} \int_0^{\Delta t} S(t) \left(1 - S(t)\right) dt \end{aligned} \tag{26}$$

which is the blur factor with the cumulative shutter function

$$S(t) = \int_0^t s(t') dt'$$

The covariance matrix diagonal elements are now the MSD curve as a function of time lag when viewed as a function of j. The covariance matrix presented in Berglund et al. [1] is recovered when $j = 1$, and it can be seen from equation 25 that it is a real symmetric band matrix where the band width increases as j increases. Following the CVE method outlined in [4], we solve for D and σ^2. There are several possibilities for this and we look at three:

$$\begin{aligned} \hat{D} &= \tfrac{1}{2j\Delta t} \overline{\Delta_{k,j}^2} + \tfrac{1}{j\Delta t} \overline{\Delta_{k,j} \Delta_{k+j,j}} \\ \hat{\sigma}^2 &= \tfrac{R}{j} \overline{\Delta_{k,j}^2} + \left(\tfrac{2R}{j} - 1 \right) \overline{\Delta_{k,j} \Delta_{k+j,j}} \end{aligned} \tag{27}$$

$$\begin{aligned} \hat{D} &= \tfrac{1}{2(j-n)\Delta t} \overline{\Delta_{k,j} \Delta_{k+n,j}}, \text{for } n = 1, ..j - 1 \\ \sigma^2 &= \tfrac{1}{2} \overline{\Delta_{k,j}^2} - \tfrac{(j-2R)}{2(j-n)} \overline{\Delta_{k,j} \Delta_{k+n,j}} \end{aligned} \tag{28}$$

$$\begin{aligned} \hat{D} &= \tfrac{1}{2(j-n)\Delta t} \overline{\Delta_{k,j} \Delta_{k+n,j}}, \text{for } n = 1, ..j - 1 \\ \hat{\sigma}^2 &= \tfrac{R}{(j-n)} \overline{\Delta_{k,j} \Delta_{k+n,j}} - \overline{\Delta_{k,j} \Delta_{k+j,j}}, \text{for } n = 1, ..j - 1 \end{aligned} \tag{29}$$

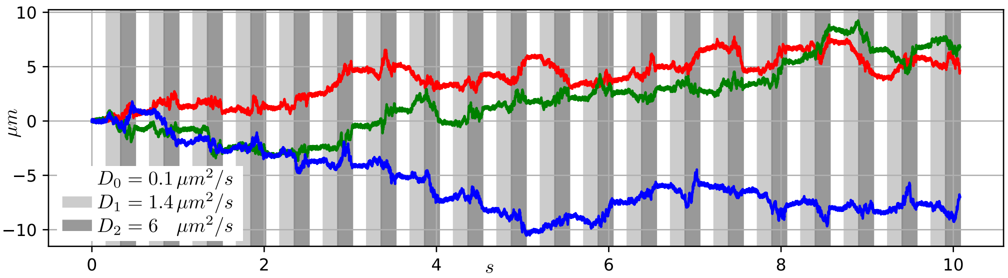

Fig. 3. **Generated trajectory for diffusion estimation test.** Trajectory coordinates for x (red), y (green) and z (blue). Each vertical section corresponds to 200 points ($168ms$) and within each section a fixed diffusion rate is maintained. The diffusion rates used are $D_0 = 0.1\mu m^2 s^{-1}$ (white), $D_1 = 1.4\mu m^2 s^{-1}$ (light gray) and $D_2 = 6\mu m^2 s^{-1}$ (dark gray). The simulation runs for 20 three-state periods giving a total of 4000 points per diffusion rate.

here hat refers to estimations and the bar is the mean taken along the diagonal of the covariance matrix, thus $\overline{\Delta^2_{k,j}} = \sum_{k=1}^{N-j} \Delta^2_{k,j}/(N-j-1) = \left\langle \Delta^2_{k,j} \right\rangle_k$, and $\overline{\Delta_{k,j}\Delta_{k+j,j}} = \langle \Delta_{k,j}\Delta_{k+j,j}\rangle_k$ where we use $\langle\rangle_k$ to indicate the mean over the parameter k. The first pair is constructed by the diagonal matrix elements for $|k-k'| = 0$ and $|k-k'| = j$. This is a direct extension of the CVE [4], and are the same when $j = 1$. For the second and third equations, we note that for $0 < |k-k'| < j$ there is no σ^2 or R dependence and thus D can be estimated directly. For σ^2 there are two choices given this D estimator. In the second pair we use the $|k-k'| = 0$ elements, and in the third pair we use $|k-k'| = j$ elements. The estimator finally used is the second pair, equation 28 , with $j = 8$ and $|k-k'| = 1$.

Comparison between the MSD, CVE and ECVE

Here we compare between the MSD, CVE and ECVE diffusion estimations. To test their performance, we simulate simple 3D diffusion where the diffusion rate is increased in steps ($D_0 = 0.1\mu m^2 s^{-1}$, $D_1 = 1.4\mu m^2 s^{-1}$ and $D_2 = 6\mu m^2 s^{-1}$) where each step is $168ms$ long. The time step used in the simulation is $0.84ms$ with a subsampling of 10 samples per time step. On top of the 3D trajectory, we add Gaussian noise with a SD of $70\mu m$ per axis. Each axis of the trajectory is shown in Fig. 3 where the colored section corresponds to a diffusion rates. Three point estimators are tested, all are related to the covariance matrix (equation 25) and are using a 16 point window for averaging. The first estimator is the MDS which is given by the mean over the diagonal elements of equation 25 when changing j and applying a linear regression to obtain D and σ^2. The second estimator is the CVE which have $j = 1$ and we solve for the D, σ^2 and take the mean over the diagonal of the covariance matrix. Lastly is the ECVE which is given by equation 6 in the main text. In Fig. 4 and Fig. 6 the three tests are shown and compared. The CVE has a poor contrast/large SD with the result that the two lower diffusion rates can hardly be distinguished. This is the drawback of using a single step where not enough difference has accumulated. Looking at the MSD, one can identify all three levels but there is a bias for slow diffusion. The ECVE shows better contrast/smaller SD compared to both the CVE and MSD and less bias. Estimating the positioning error σ^2 for the three methods (see Fig. 5 and Fig. 6) shows that the MSD has a large bias that is often negatively valued. Both the CVE and ECVE have better performance and have less bias.

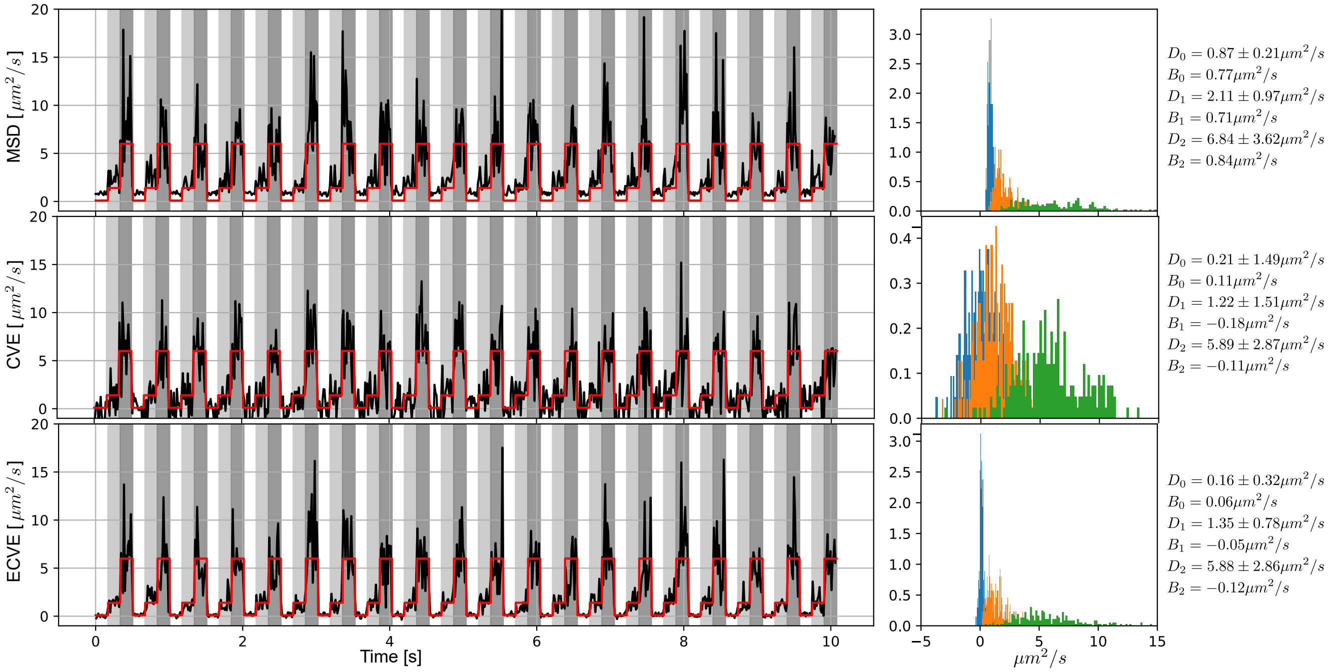

Fig. 4. **Diffuision estimation test.** Estimation of the diffusion over the trajectory shown in Fig. 3, red is the ground truth. Right plot, histograms of estimated diffusion, the sample size of each histogram is 4000 points, and is color code by the true diffusion rate; $D_0 = 0.1\mu m^2 s^{-1}$ (blue), $D_1 = 1.4\mu m^2 s^{-1}$ (orange) and $D_2 = 6\mu m^2 s^{-1}$ (green). On the right side we give the diffusion rate mean D_i with error as the distribution SD, and bias B_i which is the distance between the true value and the mean.

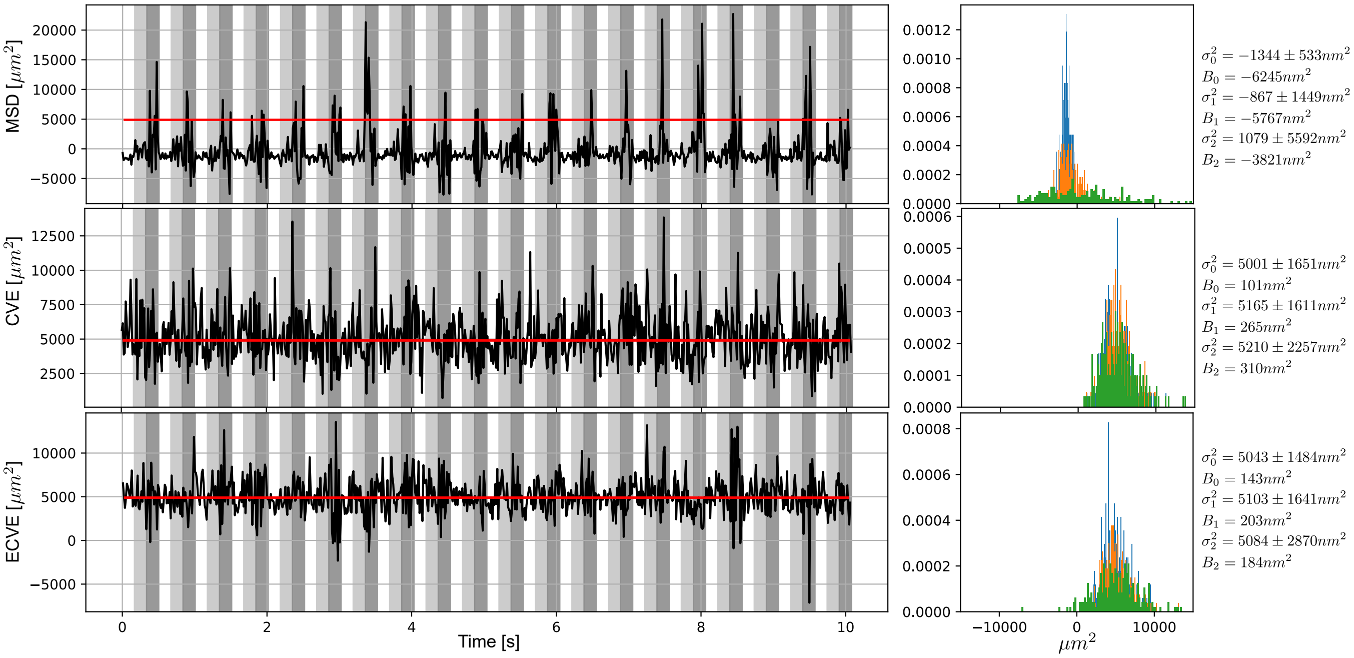

Fig. 5. **Noise estimation test.** Estimation of σ^2 for the trajectory in Fig. 3, red is the ground truth and corresponds to a SD of $70\mu m$. Right plots, histograms of estimated noise, the sample size of each histogram is 4000 points, and is color code by the true diffusion rate; $D_0 = 0.1\mu m^2 s^{-1}$ (blue), $D_1 = 1.4\mu m^2 s^{-1}$ (orange) and $D_2 = 6\mu m^2 s^{-1}$ (green). On the right side we give the estimated mean nosie σ_i^2 level with error as the distribution SD, and bias B_i which is the distance between the true value and the mean.

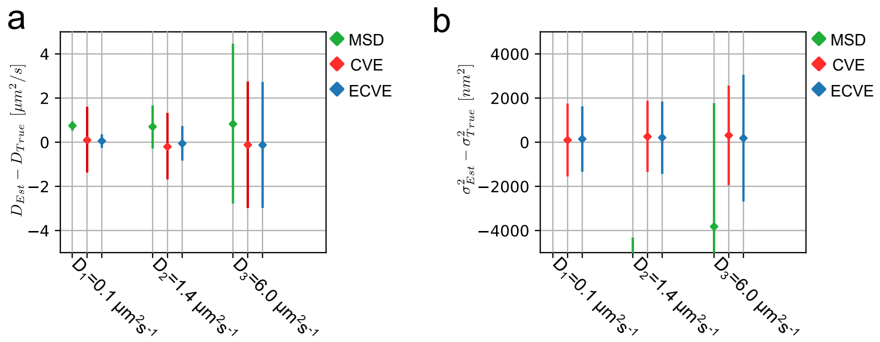

Fig. 6. **Comparason between the three diffusion estimators.** The sample size for each estimation is 4000 points and data are presented as mean ± SD. **a**, the difference between the true and estimated diffusion rates is shown for the three diffusion rates tested. Exact values can be found on the right side in Fig. 4. **b**, same as **a** plot but for the estimatied noise σ^2, here the first MSD point has a large bias and is outside of the plot range. Exact values can be found on the right side in Fig. 5.

C
Supplementary Note 3

It is possible to find an inverse to the covariance matrix, equation25, see [5], and by the spectral theorem a real symmetric matrix is diagonalizable by orthogonal matrices. Thus we can argue, as in [3], that there is a orthogonal matrix $\boldsymbol{P}$ such that $\boldsymbol{P\Sigma P}^{-1} = \boldsymbol{\Lambda}$ where $\boldsymbol{\Sigma}_j = \left\langle \boldsymbol{\Delta}_j \boldsymbol{\Delta}_j^T \right\rangle$ is the covariance matrix, equation 25, where $\boldsymbol{\Delta}_j$ is the column vector with displacement of distance j and $\boldsymbol{\Lambda}$ is a diagonal matrix with diagonal elements described by the vector $\boldsymbol{\lambda}$. Note that this also implies that there is a basis where the measurements $\boldsymbol{\Delta}_j$ are decoupled, since the diagonal matrix $\boldsymbol{\Lambda} = \boldsymbol{P\Sigma}_j\boldsymbol{P}^{-1} = \boldsymbol{P}\left\langle \boldsymbol{\Delta}_j \boldsymbol{\Delta}_j^T \right\rangle \boldsymbol{P}^T = \left\langle (\boldsymbol{P\Delta}_j)(\boldsymbol{P\Delta}_j)^T \right\rangle$. In this basis the measurements $\boldsymbol{P\Delta}_j$ are decoupled and constitute a Brownian motion with step sizes $\boldsymbol{\lambda}$. Thus, the probability for each step is given by a Gaussian, and the total log likelihood is the log of the product of all of them. Skipping all constant terms, we get for a given offset j

$$
\begin{aligned}
l(\boldsymbol{\Delta}_j) = & \quad \ln\left(\prod_i \frac{1}{\sqrt{2\pi\lambda_i}} e^{-\frac{(\boldsymbol{P\Delta}_j)_i^2}{2\lambda_i}}\right) \\
= & \quad -\frac{1}{2}\ln\left(2\pi\left|\boldsymbol{\Lambda}\right|\right) - \frac{1}{2}\left(\boldsymbol{P\Delta}_j\right)^T \boldsymbol{\Lambda}^{-1}\left(\boldsymbol{P\Delta}_j\right) \\
= & \quad -\frac{1}{2}\ln\left(2\pi\left|\boldsymbol{\Sigma}_j\right|\right) - \frac{1}{2}\boldsymbol{\Delta}_j^T \boldsymbol{\Sigma}_j^{-1} \boldsymbol{\Delta}_j
\end{aligned}
\tag{30}
$$

To include several j offsets in the likelihood, one can consider an interval of j values to use in the likelihood, $1 \leq j \leq J$ wich gives

$$
L(\{\boldsymbol{\Delta}_j\}_{j=1}^J) = \prod_{j=1}^{J} \frac{1}{\sqrt{2\pi\left|\boldsymbol{\Sigma}_j\right|}} e^{-\frac{1}{2}\boldsymbol{\Delta}_j^T \boldsymbol{\Sigma}_j^{-1} \boldsymbol{\Delta}_j}
$$

Since $\boldsymbol{\Sigma}_j$ depends on D, a maximum likelihood estimator can be obtained by finding D that maximizes the likelihood above. Since this is not a simple estimator, we will not pursue this and instead use the estimator in the main text.

D
Supplementary Note 4

A. Hidden Markov Model for estimated diffusion traces

Suppose that during a measurement, we obtain a set of measurements of the positions $\{\mathbf{r}\}$ and from these we calculate a set of diffusion constants $\{D_i\}$ using the ECVE method described in the main text. Assuming a single diffusion constant, no motion blur, and no localization error, we have a set of diffusion values $\{D_i\}$ which originates as the variance of a normal distribution with a known mean (mean is zero) but an unknown variance. The distribution of the diffusion set $\{D_i\}$ can be assumed to be inverse-Gamma distributed. Consider now a measurement sequence of M steps, $X = \{X_i\}_{i=1}^{M} = \{D_i\}_{i=1}^{M}$, in cells where the underlying dynamics is governed by an HMM model on diffusion rates. The distribution of our measured sequence, $\{D_i\}_{i=1}^{M}$, is thus a mixture of inverse-Gamma distributions, with parameters $\phi = \{\phi_j\}$, corresponding to different states $S = \{S_j\}$ where $j \in \{1, ..., N\}$ iterates over the N different states. The relation between the states is given by an $N \times N$ transition matrix $\boldsymbol{a}$ with elements a_{kl} which are the probability of moving from state k to state l with the normalization condition $\sum_l a_{kl} = 1$. Using these we can construct the likelihood of obtaining a sequence of measurements for the unknown parameters $\gamma = \{\boldsymbol{a}, S, \phi\}$. Now, the goal of the game is to try to find the parameters that maximize the likelihood of the measured sequence. Note that if we did know the state S_j for each measurement X_i we would be able to organize our data in pairs $\{(X_i, S_j)\}_{i=1}^{M}$ which indicates exactly which data point belong to which state and thus which distribution. The parameter ϕ_j for each distribution can be calculated separately and thus the probabilities $P(X_i|S_j)$ can be obtained from each inverse-Gamma distribution. The likelihood of obtaining a sequence $A = \{a_{S_{i-1}S_i}\}_{i=1}^{M}$ from an unknown transition matrix $\boldsymbol{a}$ is then given by

$$L(A|X, S, \phi) = P(S_1) \prod_{i>1} P(X_i|S_i, \phi) \cdot P(S_i|S_{i-1})$$

where $P(S_i|S_{i-1}) = a_{S_{i-1}S_i}$, with $P(S_1)$ as the probability of starting at state S_1. The problem is thus reduced to maximizing a multidimensional first degree polynomial. Unfortunately the state sequence is not known and are thus considered hidden, to account for this we marginalize over the state S, and the likelihood becomes

$$L(\gamma|X) = \sum_S L(A, \phi|X, S) = \sum_S P(S_1) \prod_{i>1} P(X_i|S_i) \cdot P(S_i|S_{i-1})$$

finding $\hat{\gamma} = argmax_\gamma L(\gamma|X)$ is a much harder problem, where numerical search is required. For this task there are several packages available, here we use the python Pomegranate HMM suite [6].

B. Gamma and inverse-Gamma distribution relation

Within the Pomegranate HMM suite, the inverse-Gamma distribution is not available but the Gamma distribution is. Fortunately, the two are closely related. The gamma distribution is given by

$$G(x) = \frac{\beta^\alpha}{\Gamma(\alpha)} \cdot x^{\alpha-1} \cdot e^{-\beta\cdot x} = \frac{1}{\beta \cdot \Gamma(\alpha)} \cdot (\beta \cdot x)^{\alpha-1} \cdot e^{-\beta\cdot x}$$

where $\bar{x} = \alpha/\beta$, $\sigma_x^2 = \alpha/\beta^2$. We can relate the Gamma distribution to the inverse-Gamma by the transformation $y = 1/x$, from probability theory we have that $G_y(y) = G_x(1/y) \cdot J(x = 1/y)$, where J is the Jacobian of the transformation. This gives

$$G_x(1/y) = \frac{\beta^\alpha}{\Gamma(\alpha)} \cdot y^{-\alpha+1} \cdot e^{-\frac{\beta}{y}} \cdot \frac{1}{y^2} = \frac{\beta^\alpha}{\Gamma(\alpha)} \cdot y^{-\alpha-1} \cdot e^{-\frac{\beta}{y}} = G_{inv}(y)$$

Thus, getting the parameters for the inverse-Gamma distribution is the same as the ones for the Gamma with inverted data. Finally, there is a slightly different interpretation of the parameters: β is the rate parameter in the Gamma distribution but is the scale parameter in the inverse-Gamma distribution.

C. Handling negative values when fitting inverse-gamma distributions

For all three estimators tested, MSD, ECV and ECVE, one might get negative values. These occur when evaluating diffusion close to zero and will be affected by the noise level in the signal. The negative values will be less when using higher offset j compare to $|k - k'|$, but this also depends on the dynamics analyzed. One way to handle negative values is to move to higher offset values, j, or average over a larger interval. However, this is not desirable if the goal is analysis of fast dynamics. For a Gaussian distribution with known mean and unknown variance, the most apt distribution over the unknown variance is inverse-Gamma. Unfortunately, the inverse-Gamma is a distribution for positive values, and it would be preferable to be able to handle small negative values within the inverse-Gamma model. The way we handle this is to shift all values by a bias to

the positive axis. We note that if we shift the data by the amount b such that the data is $x+b$ we have that $\overline{x+b} = \overline{x} + b$ and that $\sigma^2_{x+b} = \sigma^2_x$.

The inverse gamma distribution is given by

$$G_{inv}(x) = \frac{\beta^\alpha}{\Gamma(\alpha)} \cdot x^{-\alpha-1} \cdot e^{-\frac{\beta}{x}}$$

where $\bar{x} = \beta/(\alpha-1)$, $\sigma^2_x = \beta^2/(\alpha-1)^2(\alpha-2)$ if $\alpha > 2$. Thus this means that if we get parameters α' and β' for the $\{x+b\}$ data we get $\overline{x+b} = \beta'/(\alpha'-1) = \overline{x} + b = \beta/(\alpha-1) + b$ and that $\sigma^2_{x+b} = \beta'^2/(\alpha'-1)^2(\alpha'-2) = \beta^2/(\alpha-1)^2(\alpha-2)$ where α and β are the non shifted parameters. This gives the relations between shifted and non shifted data:

$$\alpha = \frac{(\overline{x+b} - b)^2}{\sigma^2_{x+b}} + 2$$

$$\beta = (\overline{x+b} - b)(\alpha - 1)$$

We note that by shifting the inverse-Gamma a positive amount, we are forcing the distribution to be more Gaussian like. One is thus losing the skewness of the inverse-Gamma distribution. The skewness is an important property that we desire to preserve. Thus, the shift must be relatively small. On the other hand, if no shift is added we allow for distributions with infinite variance. By adding a shift, the variance is suppressed. A shift of $0.01 - 10\mu m^2 s^{-1}$ gives good results and for most data we used a shift of $2\mu m^2 s^{-1}$. The exception is for *E. coli* Trigger Factor data measured with the pattern which has a missing center shot and is presented in sub.sec.(H), for this data we used a shift of $5\mu m^2 s^{-1}$.

D. Bootstrap sampling for error estimation

To estimate error in the HMM parameters for simulations and TF data, we did a bootstrap sampling over the trajectories. From the pool of trajectories we draw with replacement the same number of trajectories as there are in the pool. The new trajectory set is analyzed with the HMM analysis. This is done 100 times, giving us a spread in the HMM parameter estimation.

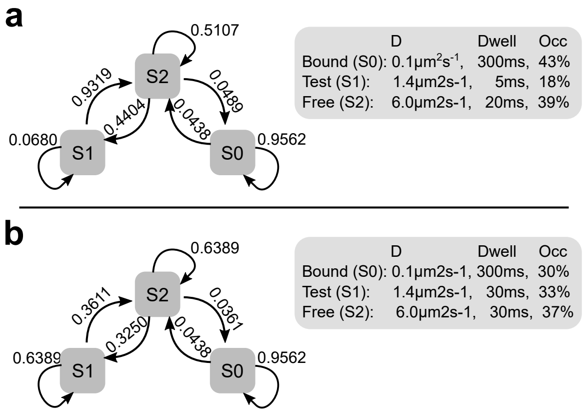

Fig. 7. **Parameters of the HMM models used.** HMM model parameters for simulated trajectories in *E. coli* like geometry, transition probabilities are for a 13.44ms which corresponds to the time window of 16 localizations. **a** is the model with a short-lived intermediate state. **b** is a more relaxed model where the free and intermediate state have both a dwell time of 30ms.

E
Supplementary Note 5

Simulations are performed by setting up a diffusion HMM model with 3 states characterized by the transition matrix shown in Fig. 7. From this, we generate Viterbi paths where the length is selected at random within the interval $50ms$ and $800ms$. The Viterbi paths are then used for creating particle trajectories where the x,y,z step sizes are sampled from a gaussian with the proper diffusion rate. At this stage we have an unbounded diffusion. By folding this trajectory within a cell structure using reflective boundary conditions, we obtain a diffusion ground truth path within a cell with a time resolution of $20\mu s$. For diffusion analysis of simulated trajectories, we used the ECVE with a time lag of $j = 8$ with a 16 point localization time window, see Fig. 8**b**. In the main text we present the results when simulating 100 trajectories using the model in Fig. 7**a**. We see that the fitted HMM has a hard time extracting the correct parameters for the diffusion rates and occupancies. This also holds for the dwell times which are poorly estimated.

In Fig. 8 we present results of the second simulation based on the 3-state HMM in Fig. 7**b**. For this model 50 trajectories are simulated in an *E. coli* like geometry. The diffusion estimations from the reconstructed trajectories are fitted to a 3-state HMM assuming inverse-gamma distributions as emission distributions. In Fig. 8**b**, the red trace is the predicted Viterbi path, and in Fig. 8**c**, we show the distribution of all estimated diffusion values together with the predicted inverse-gamma distributions from the trained HMM. As before we see that the histogram does not correctly reflect the fast state. Instead, we see a mean value between the fast and the intermediate state. Two major distributions can be identified in the histogram: the slow with mean diffusion $0.26 \pm 0.03\mu m^2 s^{-1}$ (ground truth $0.1\mu m^2 s^{-1}$), with standard error from bootstrapping, and occupancy $33 \pm 8\%$ (ground truth 30%) and the fast state with mean diffusion $3.18 \pm 0.26\mu m^2 s^{-1}$ (ground truth $6\mu m^2 s^{-1}$) and occupancy $32 \pm 8\%$ (ground truth 37%). The intermediate state is identified by the HMM analysis in between the two distributions with a $36 \pm 8\%$ (ground truth 33%) occupancy and mean diffusion $1.45 \pm 0.24\mu m^2 s^{-1}$ (ground truth $1.4\mu m^2 s^{-1}$). Here, the occupancies are here better estimated, and the dwell times are also better represented with $373ms$ ($300ms$) for the bound state, $44ms$ ($30ms$) for the intermediate state and $47ms$ ($30ms$) for the fast state, where the ground truth is reported within parentheses. The estimated parameters are better but the predicted Viterbi path is still not capturing the faster transitions of a trajectory.

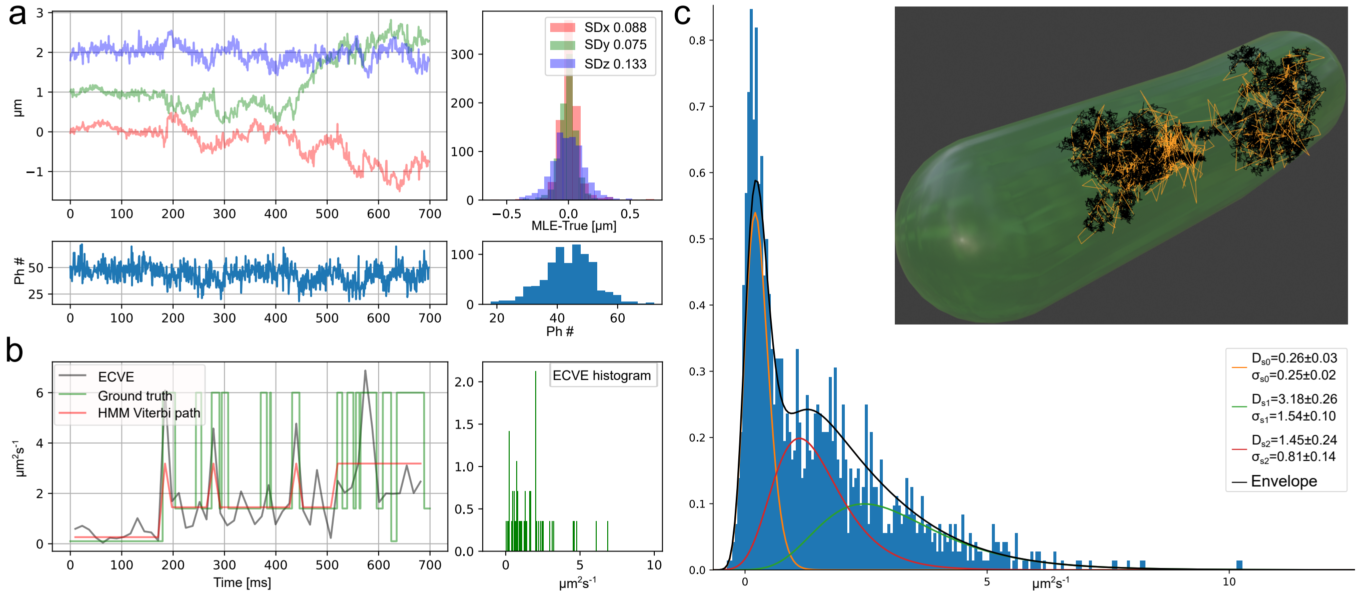

Fig. 8. **Tracking simulation.** Example trajectory with HMM analysis of all 50 simulated trajectories of the model in Fig.(7**b**). **a**, upper left; example trajectory with x (red) y (green) z (blue) estimated position coordinates with corresponding photon counts (bottom). Upper right; the deviation away from ground truth with the SD of the error distribution in the legend, (bottom) is the photon count distribution. **b**, left; diffusion point estimation (gray) along the trajectory in **a**, ground truth diffusion is in green, and the HMM prediction in red. Right; the distribution of estimated diffusions over the trajectory in **a**. **c**, distribution of all diffusion estimates from the 50 trajectories. The 3 state HMM model prediction of the emission distributions are plotted on top of the histogram. The inverse-gamma mean and SD with standard error of each emission distribution is reported in the legend. Inset, 3D representation of the cell volume with ground truth trajectory in black and the estimated path from **a** in yellow. Source data are provided as a Source Data file.

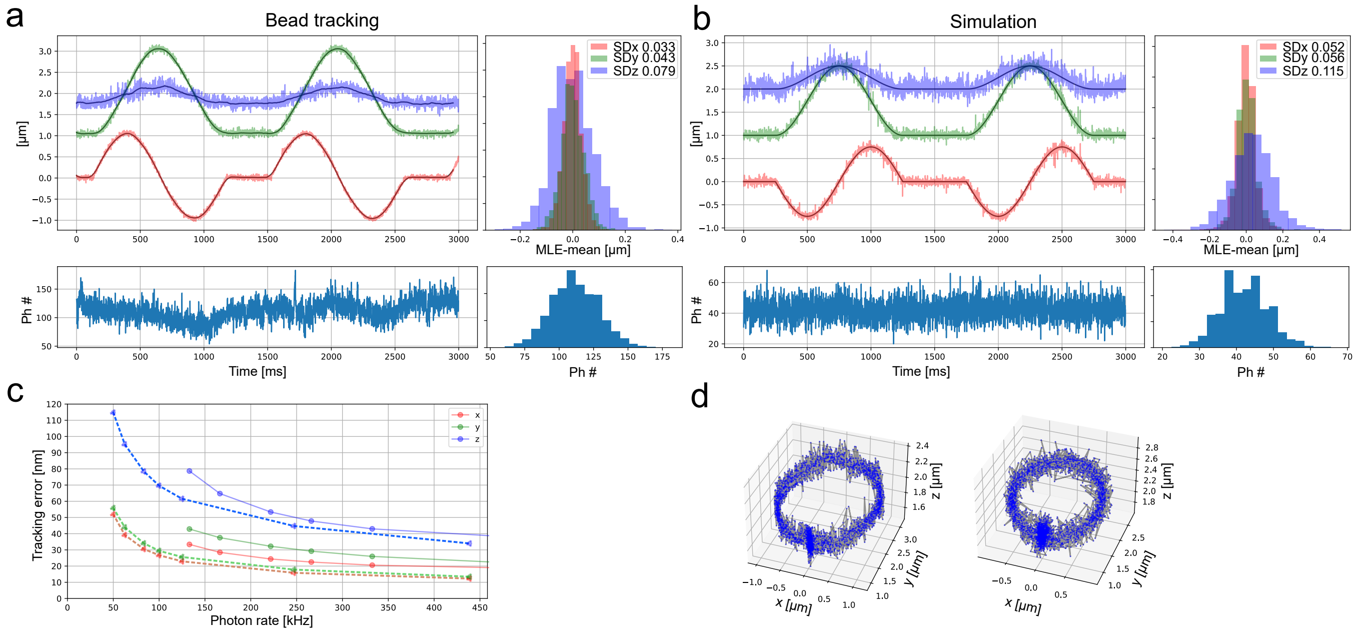

Fig. 9. **Resolution test at a higher photon count rate.** **a**, Tracking of beads fixed in agarose, the piezo stage is programmed to trace circles with a x,y-radius of $1\mu m$ and a z peak to peak oscillation of $0.5\mu m$. Upper left; Trajectory estimated x (red), y (green) and z (blue) coordinates with a temporal resolution of $0.84ms$. Upper right; distribution of SD over the trajectory between estimation and a moving average with a window size of 100 points. Bottom left; photon counts per localization which corresponds to 60kHz photon count rate. To the right the photon count distribution. **b**, Similar to **a** but for a simulated trajectory with an x,y-radius of $0.75\mu m$ and a z peak to peak oscillation of $0.5\mu m$, the error distribution (upper right) is here the distance to ground truth. **c**, resolution as a function of photon count rate for x (red), y (green) and z (blue), solid lines are for the bead data and dashed lines correspond to the simulations. Each point is the SD of the distance histogram in **a** and **b**. These are obtained by increasing the exponential moving average of the likelihood. **d**, 3D point scatter plot of the estimated (left) and simulated (right) trajectory. Source data are provided as a Source Data file.

F
Supplementary Note 6

Using an experimentally measured PSF z-stack that has been interpolated down to voxels of 10nm, we simulate the microscope laser pattern with an added gaussian position noise of 10nm for xy and 10nm for z. The gaussian noise is added since characterization of the beam profile after reflection on a reflective sample indicates a small beam/sample movement at high frame rates (200fps) when analyzing images taken from a EMCCD. At low frame rate, this is averaged out; however, for tracking at 1ms time scale, this must be taken into account.

From the PSF stack we sample the PSF intensities and generate photon counts assuming Poissonian statistics. The real-time feedback loop for the piezo stage is simulated as a long moving average over the ground truth data. This limits the speed of the simulated piezo-stage to behave close to what is observed in experiments. The output of the simulation is of the same format as a real experiment and thus can be analyzed by the same pipeline as experimental acquired data.

A. Circular motion

Validation of the tracking is done by comparing simulation and real-time tracking of beads. For these we simulated similar rotational motion and at photon counts similar to experiments. A comparison between tracking of beads and simulations is shown in main Fig. 2 and a measurement at higher photon rate is shown in Fig. 9.

To see more clearly the stage behavior, we can take a moving average of the likelihood as described in Methods Likelihood exponential averaging section. With the exponential parameter set to 0.4, we get an effective time resolution of $1.98ms$. In Fig. 10 we show the results of the trajectory reconstruction when tracking a bead while the stage is following circle. Upon a closer look at Fig. 10**a**, where we have added a 20 point sliding window to give a clearer view, we can identify oscillations and stage corrections. We attribute these artifacts to the stage feedback system and vibrations induced by the movement. To further investigate, we looked at stationary beads to see if the stage feed-back system is inducing any detectable movement while not moving. To test this we compared tracking of stationary beads while the stage is either in the close loop or open loop configuration, see Fig. 11. No obviouse differenc is detected, but what can be noted is that at this temporal resolution and photon coutunt we have a lower limit on the diffusion detection.

B. Cramer-Rao lower bound

To estimate the CRLB we need to calculate the right side of equation 17, plugging in the negative log likelihood, equation 7, into the equation and using $\lambda_i = c \cdot \Phi_i(\mathbf{r}_p)$ we obtain

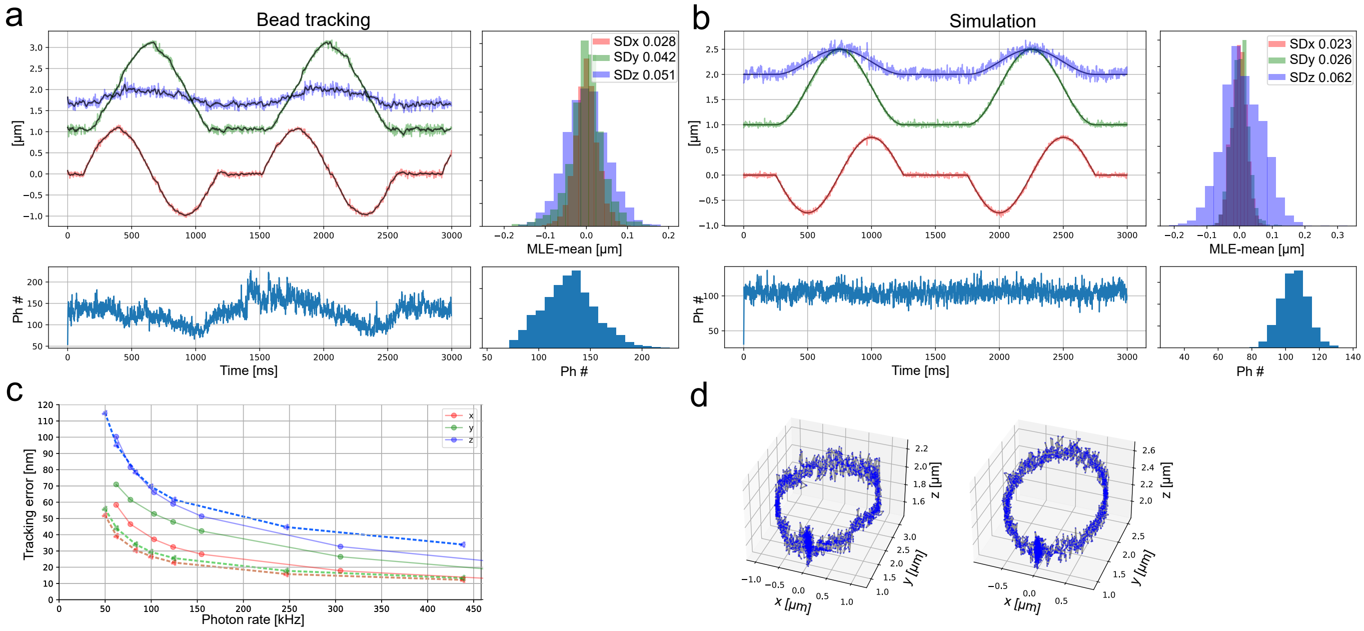

Fig. 10. **Resolution test with sliding window averaging.** Resolution test when taking a moving average during trajectory reconstruction giving an effective time resolution of $1.98ms$. **a**, Tracking of beads fixed in agarose, the piezo stage is programmed to follow circles with a x,y-radius of $1\mu m$ and a z peak to peak oscillation of $0.5\mu m$. Upper left; Trajectory estimated x (red), y (green) and z (blue) coordinates with a $0.84ms$ between localizations. Upper right; distribution of SD over the trajectory between estimation and a moving average with a window size of 20 points. Bottom left; photon counts per localization which corresponds to 60kHz photon count rate. To the right the photon count distribution. **b**, Similar to **a** but for a simulated trajectory with an x,y-radius of $0.75\mu m$ and a z peak to peak oscillation of $0.5\mu m$, the error distribution (upper right) is here the distance to ground truth. **c**, resolution as a function of photon count rate for x (red), y (green) and z (blue), solid lines are for the bead data and dashed lines correspond to the simulations. Each point is the SD of the distance histogram in **a** and **b**.These are obtained by increasing the exponential moving average of the likelihood. **d**, 3D point scatter plot of the estimated (left) and simulated (right) trajectory. Source data are provided as a Source Data file.

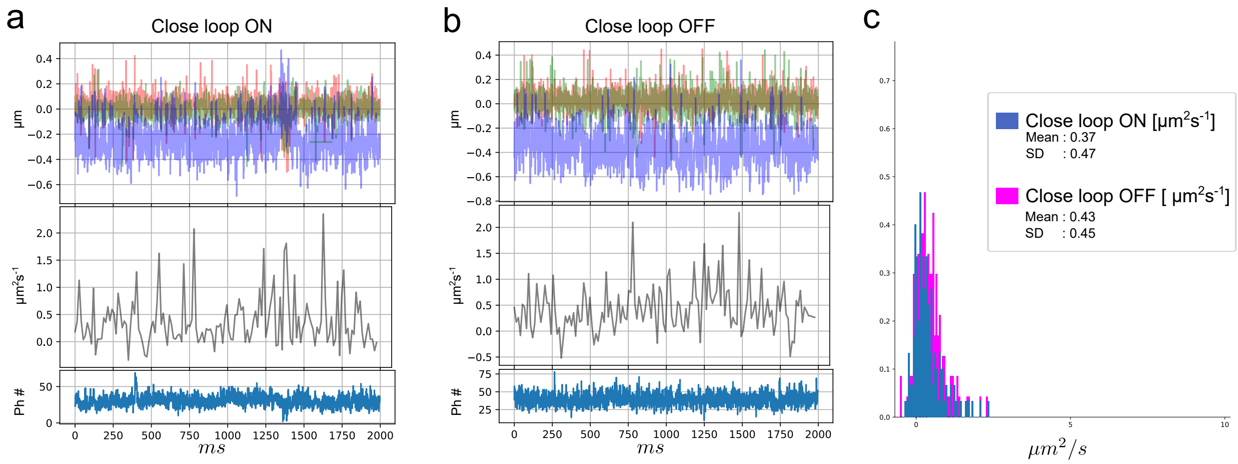

Fig. 11. **Resolution test when taking stationary beads**. **a**, Tracking of beads fixed in agarose, while the nanoMAx stage is set in close loop configurtion. The trajectory length is 2 seconds and shown is the x (red), y (green) and z (blue) position in the top plot, the estimated diffusion in the middle and the photon counts at the bottom. **b**, similar to **a** but with the nanoMax in the open loop configuration. **c**, the diffusion histograms of the close and open loop nanoMax configuration. Source data are provided as a Source Data file.

$$var(x) \geq \frac{1}{c \cdot \sum_i \Phi_i \cdot \left(\frac{\Phi_i}{\sum_i \Phi_i}\right)^{-2} \left(\left(\frac{\partial}{\partial x}\left(\frac{\Phi_i}{\sum_i \Phi_i}\right)\right)^2 - \frac{\Phi_i}{\sum_i \Phi_i}\frac{\partial^2}{\partial x^2}\left(\frac{\Phi_i}{\sum_i \Phi_i}\right)\right)}$$

As seen this depends on the first and second derivative of the PSF ratios and c which represent the tracked reporter response and is directly related to the photon counts. To calculate the first and second derivative we use the Sobel–Feldman operator which includes a simple difference, $h\acute{}(-1) = 1, h\acute{}(0) = 0, h\acute{}(1) = -1$, in the derivative direction and a smoothing,

$h(-1) = 1, h(0) = 2, h(1) = 1$, in the same direction. To construct the Sobel–Feldman operator, $H_d^{(D)}$, for dimensions D in the direction d, one do

$$H_{x_d}^{(D)}(x_{,1},..,x_D) = h\acute{}(x_d)\prod_{j=2}^{D} h(x_j) \tag{31}$$

where $x_j \in [-1, 0, 1]$. The dimensionality of $H_d^{(D)}$ will be $3^{\otimes D}$. We are after the three operations $H_x^{(3)} = H_x$,$H_y^{(3)} = H_y$ and $H_z^{(3)} = H_z$. Using equation 31 we get the three matrices

$$H_x(:,:,-1) = \begin{pmatrix} 1 & 0 & -1 \\ 2 & 0 & -2 \\ 1 & 0 & -1 \end{pmatrix}, H_x(:,:,0) = \begin{pmatrix} 2 & 0 & -2 \\ 4 & 0 & -4 \\ 2 & 0 & -2 \end{pmatrix}, H_x(:,:,1) = \begin{pmatrix} 1 & 0 & -1 \\ 2 & 0 & -2 \\ 1 & 0 & -1 \end{pmatrix}$$

$$H_y(:,:,-1) = \begin{pmatrix} 1 & 2 & 1 \\ 0 & 0 & 0 \\ -1 & -2 & -1 \end{pmatrix}, H_y(:,:,0) = \begin{pmatrix} 2 & 4 & 2 \\ 0 & 0 & 0 \\ -2 & -4 & -2 \end{pmatrix}, H_y(:,:,1) = \begin{pmatrix} 1 & 2 & 1 \\ 0 & 0 & 0 \\ -1 & -2 & -1 \end{pmatrix}$$

$$H_z(:,:,-1) = \begin{pmatrix} 1 & 2 & 1 \\ 2 & 4 & 2 \\ 1 & 2 & 1 \end{pmatrix}, H_z(:,:,0) = \begin{pmatrix} 0 & 0 & 0 \\ 0 & 0 & 0 \\ 0 & 0 & 0 \end{pmatrix}, H_z(:,:,1) = \begin{pmatrix} -1 & -2 & -1 \\ -2 & -4 & -2 \\ -1 & -2 & -1 \end{pmatrix}$$

Appling the Sobel-Feldman operation, H_j, to the PSF ratio $S_i = \Phi_i / \sum_i \Phi_i$is done by a 3D convolution, $H_j * S_i$. To do this we can utilize the convolution theorem that states that the convolution of f and g can be done as a multiplication in fourier space, mathematically this is given by

$$f * g = \mathcal{F}^{-1}\{\mathcal{F}\{f\} \cdot \mathcal{F}\{g\}\}$$

For the fourier transform we use the numpy N-dimensional discrete fast Fourier transform. We evaluate the CRLB for the PSF and pattern used for the bead data in Fig. 2, the c value used is set as the mean of the estimated c-value after 100 estimations. This choice of c-value gives a representative CRLB for the bead tracking which has an average photon count rate of $60kHz$. The CRLB is calculated for x, y and z and we then take the mean over these to obtain a 3D CRLB map. The results are shown in Fig. 12, here three planes of the 3D volume are shown together with a plot of the x,y and z axis passing through the center of the volume. This shows that the CRLB is uniform within the volume with an increase closer to the edges of the volume.

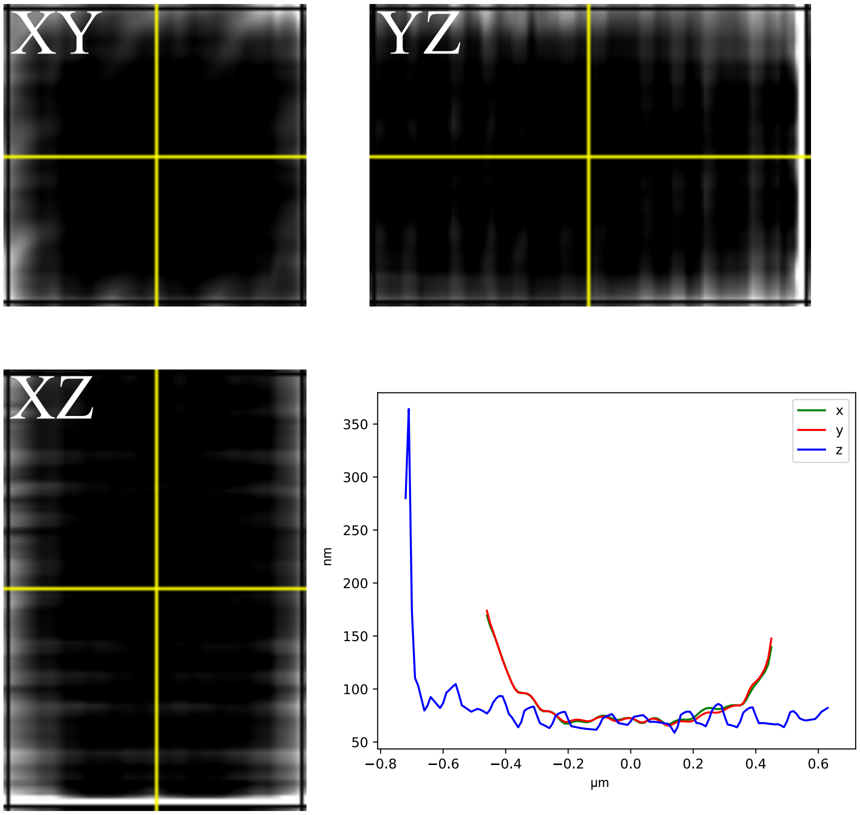

Fig. 12. **Cramer-Rao lower bound estimate over the volume of the PSF pattern.** The three panels of the CRLB are shown, these correspond to the planes; xy, yz and xy. The yellow lines indicate how the three cuts are related to each other. Bottom right, shows the CRLB plot through the center for x,y, and z. Source data are provided as a Source Data file.

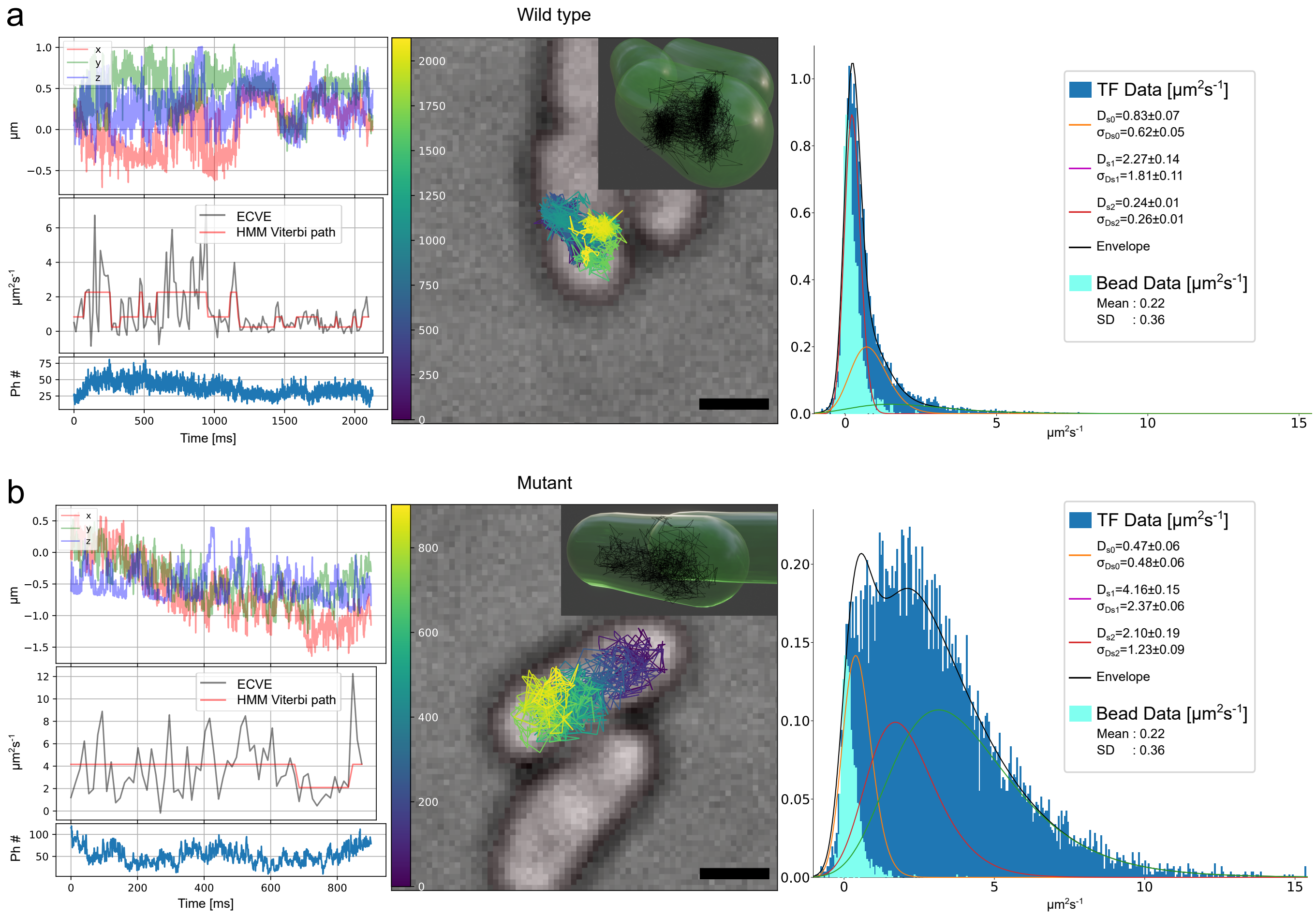

Fig. 13. **Tracking TF in WT and mutant E. Coli cells.** After the microscope calibration we conducted two experiments during the same day: one experiment for the wild-type where 277 trajectories were acquired from 134 cells, and a second experiment for the mutant 538 trajectories were acquired from 65 cells. For each sample type, wild-type **a** and mutant **b**, an example trajectory (left) is shown together with the distribution of the accumulated diffusion histograms over all measured trajectories of each type (right side). Each horizontal block shows; Center image is the widefield cell image (black bar $1\mu m$) with estimated trajectory color coded over time. Inset is the 3D trajectory with a cell membrane representation based on the widefield image. Left plots, for the same trajectory shown in the center image, are from the top; position estimation, point wise diffusion estimation (gray) with HMM Viterbi path (red) and photon counts in the bottom plot. Right plot; in dark blue is the histograms over all diffusion estimates acquired for the WT or Mutant together with the HMM emission distributions. The light blue overlaid histogram is the diffusion estimated distribution for stationary beads with the nanoMax stage in the closed loop configuration, this indicates the lower detection limit at this photon count rate. In the histogram legend are the mean diffusion values and SD for each HMM estimated distribution with standard error from bootstrapping, and for the bead data is the mean and SD of the light blue distribution. Source data are provided as a Source Data file.

G
Supplementary Note 7

To test that the TF measurements are reproducible we made a repeat experiment of the measurements presented in the main article. The results are shown in Fig. 13. For the WT the three state HMM the diffusion rates found are $D_{s0} = 0.83 \pm 0.07\mu m^2 s^{-1}$, $D_{s1} = 2.27 \pm 0.14\mu m^2 s^{-1}$ and $D_{s2} = 0.24 \pm 0.01\mu m^2 s^{-1}$ with corresponding occupancies; $O_{S0} = 30 \pm 2\%$, $O_{S1} = 11 \pm 2\%$ and $O_{S2} = 59 \pm 3\%$. Comparing this to Fig. 4 in the main text we see that the distribution and the found diffusion states are about the same. For the mutant we obtian the diffusion rates $D_{s0} = 0.47 \pm 0.06\mu m^2 s^{-1}$, $D_{s1} = 4.16 \pm 0.15\mu m^2 s^{-1}$ and $D_{s2} = 2.10 \pm 0.19\mu m^2 s^{-1}$ with the corresponding occupancies $O_{S0} = 0.17 \pm 0.03\%$, $O_{S1} = 0.55 \pm 0.05\%$ and $O_{S2} = 0.28 \pm 0.04\%$. A comparasone with Fig. 4 and data in the main text show that the mutant also have the same behavior as the first measurement.

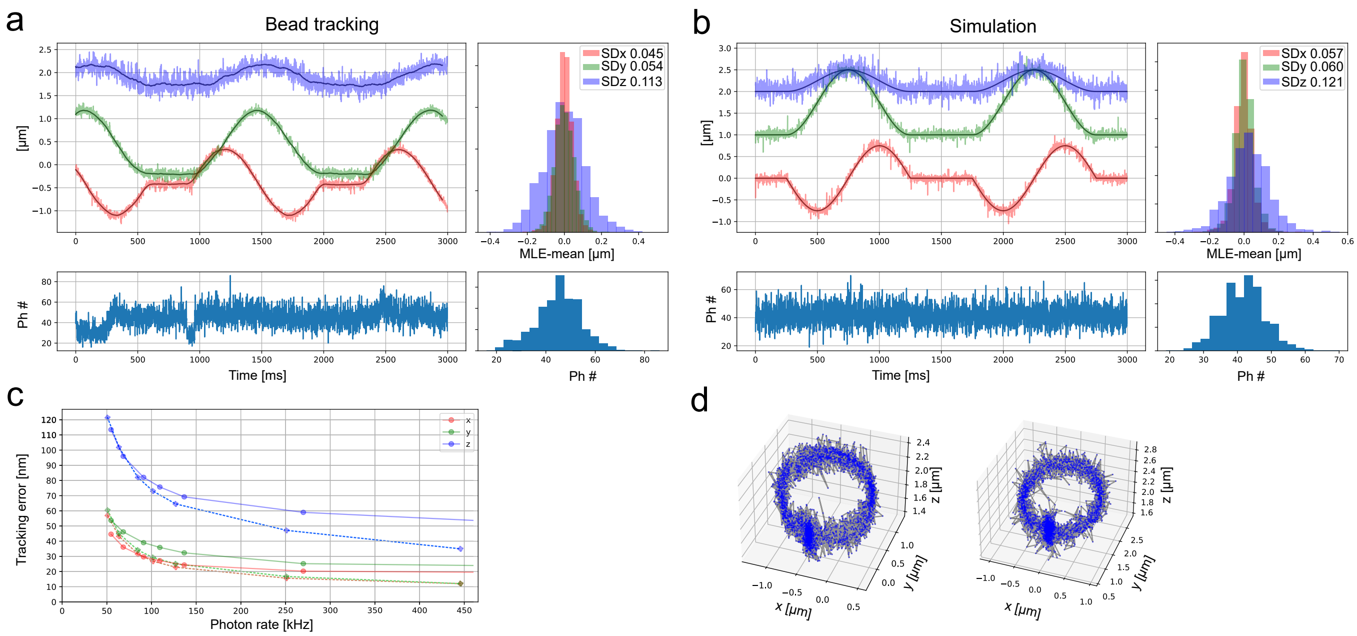

Fig. 14. **Resolution test when removing the center shot in the tracking pattern.** **a**, Tracking of beads fixed in agarose, the piezo stage is programmed to trace circles with a x,y-radius of $1\mu m$ and a z peak to peak oscillation of $0.5\mu m$. Upper left; Trajectory estimated x (red), y (green) and z (blue) coordinates with a $0.82ms$ between localizations (41 shots in this pattern). Upper right; distribution of SD over the trajectory between estimation and a moving average with a window size of 100 points. Bottom left; photon counts per localization which corresponds to 60kHz photon count rate. To the right the photon count distribution. **b**, Similar to **a** but for a simulated trajectory with an x,y-radius of $0.75\mu m$ and a z peak to peak oscillation of $0.5\mu m$, the error distribution (upper right) is here the distance to ground truth. **c**, resolution as a function of photon count rate for x (red), y (green) and z (blue), solid lines are for the bead data and dashed lines correspond to the simulations. Each point is the SD of the distance histogram in **a** and **b**.These are obtained by increasing the exponential moving average of the likelihood. **d**, 3D point scatter plot of the estimated (left) and simulated (right) trajectory. Source data are provided as a Source Data file.

H
Supplementary Note 8

The theory predicts that higher resolution can be obtained by moving the Gaussian beams further apart, see Supplementary Note 1. This means that removing the center position in our pattern should increase the tracking resolution when keeping the same photon counts. To test this we did all the measurements with a pattern configuration where the center shot is removed, in practice this means that there is a hole in the center of our pattern. The resolution test in Fig. 14 shows that the simulation loses resolution compared to Fig. 9. What can be observed is that the pattern with a hole in the center has more sporadic jumps in the reconstruction indicating that the removal of the center lowers the informational content and thus lowers the noise tolerance. The bead tracking experiment does indicate a small resolution increase in the lateral direction but this is marginal. Looking at higher averaging (Fig. 15) we see, as before, oscillations when tracking while the stage is moving, again we attribute these to the stage feedback-loop and induced vibrations during stage movement.

As for the original pattern, we used the new pattern to simulate on an *E. coli*-like geometry and experimental measurements on the *E. coli* Trigger Factor. Results of the simulation when generating 100 trajectories according to the HMM model apper in Fig. 7**a** and use the same analysis as for the data reported in the main text. The diffusion estimations from the reconstructed trajectories are fitted to a 3-state HMM assuming inverse-gamma distributions as emission distributions. In Fig. 16**b**, the red trace is the predicted Viterbi path, and in Fig. 16**c**, we show the distribution of all estimated diffusion values together with the predicted inverse-gamma distributions from the trained HMM. As before we see that the histogram does not correctly reflect the fast state, we instead see a mean value between the fast and the intermediate state. Two major distributions can be identified in the histogram: the slow with mean diffusion $0.21 \pm 0.02\mu m^2 s^{-1}$ (ground truth $0.1\mu m^2 s^{-1}$), with standard error, and occupancy $41 \pm 7\%$ (ground truth 43%) and the fast state with mean diffusion $2.90 \pm 0.05\mu m^2 s^{-1}$ (ground truth $6\mu m^2 s^{-1}$) and occupancy $42 \pm 4\%$ (ground truth 38%). The intermediate state is identified by the HMM analysis in between the two distributions with a $17 \pm 5\%$ (ground truth 18%) occupancy and mean diffusion $0.60 \pm 0.14\mu m^2 s^{-1}$ (ground truth $1.4\mu m^2 s^{-1}$). As seen, the results are similar to those presented with the full pattern. The occupancy is about right, the slow state is clear in the histogram, but we see an average of the fast and intermediate state for the diffusion rate.

Measurements on *E. coli* Trigger Factor were also done using the pattern with a hole at the center. In Fig. 17 the results are presented; both are selected to show the dynamical behavior of the WT and mutant TF and for that reason, they are longer than the average trajectory. In the case of the wild-type, see Fig. 17**a**, we frequently observe a bound state at low diffusion which is interrupted by occasional periods of high diffusion. All trajectories are fitted to a 3-state HMM, see Fig. 17**a** right side where

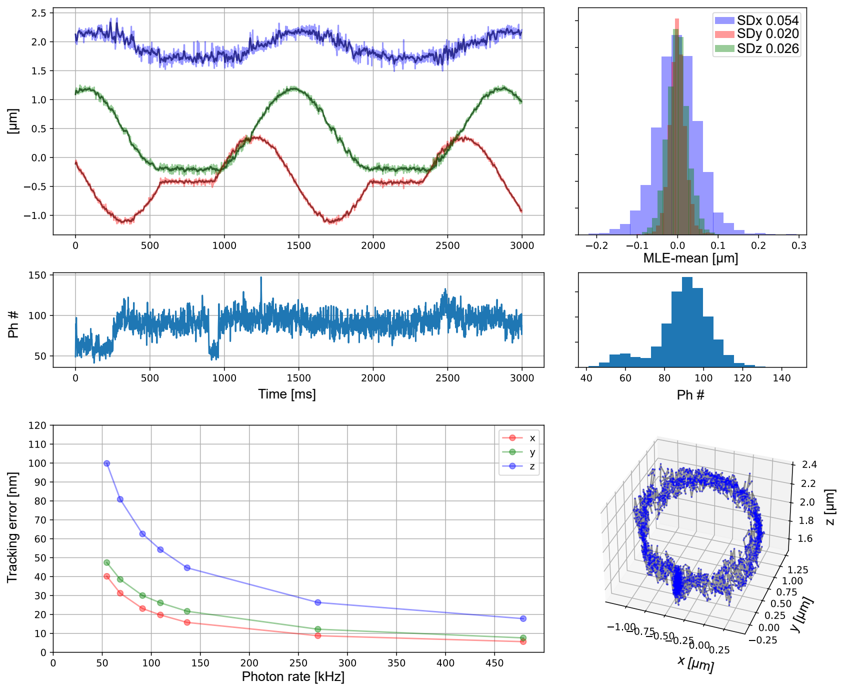

Fig. 15. **Tracking beads fixed in agarose with likelihood average.** The piezo stage was programmed to follow 1Hz circles of x,y-radius of $1\mu m$ and a z peak to peak oscillation of $0.5\mu m$. During reconstruction a moving average is used with the averaging parameter 0.4 giving an effective time resolution of $1.93ms$. Upper left; Trajectory estimated x (red), y (green) and z (blue) coordinates. Clear oscillations can be seen in the x and y trajectory. Upper right; distribution of SD over the trajectory between estimation and a moving average with a window size of 10 points. Middle left; photon counts per localization and to the right its distribution. Bottom left; resolution as a function of photon rate for x (red), y (green) and z (blue). Obtained by increasing the exponential moving average of the likelihood. Bottom right; 3D point scatter plot of the estimated trajectory. Source data are provided as a Source Data file.

the identified HMM is plotted together with the diffusion histogram. The occupancies for the bound ($0.43 \pm 0.05\mu m^2 s^{-1}$) and the intermediate ($1.20 \pm 0.15\mu m^2 s^{-1}$) states are $21 \pm 5\%$ and $48 \pm 2\%$ respectively and constitute the majority of the events. The parantheses and the HMM errors are from bootstraping over trajectories 100 times. The remaining $30 \pm 5\%$ of the time, the TF is in a more free state ($2.80 \pm 0.14\mu m^2 s^{-1}$) exploring a larger volume. Looking at the mutant, we see a very different situation, see Fig. 17**b**. A bound state ($1.02 \pm 0.09\mu m^2 s^{-1}$) is found by the HMM, with an occupancy of $22 \pm 6\%$. The mutant's intermediate state ($2.36 \pm 0.12\mu m^2 s^{-1}$) is here as fast as the wild-type's free state and is occupied $38 \pm 6\%$ of the time. As before we see in the histogram a large tail at higher diffusion which the HMM assigns to a fast ($4.60 \pm 0.20\mu m^2 s^{-1}$) state with an occupancy of $40 \pm 6\%$. It is clear that the mutant's mobility is much higher than that of the wild-type, allowing the mutant TF to explore larger parts of the *E. coli* cell. In conclusion the diffusion speeds available for the mutant TF are higher than those allowed for the wild-type TF which was also seen with the tracking pattern with the center shot.

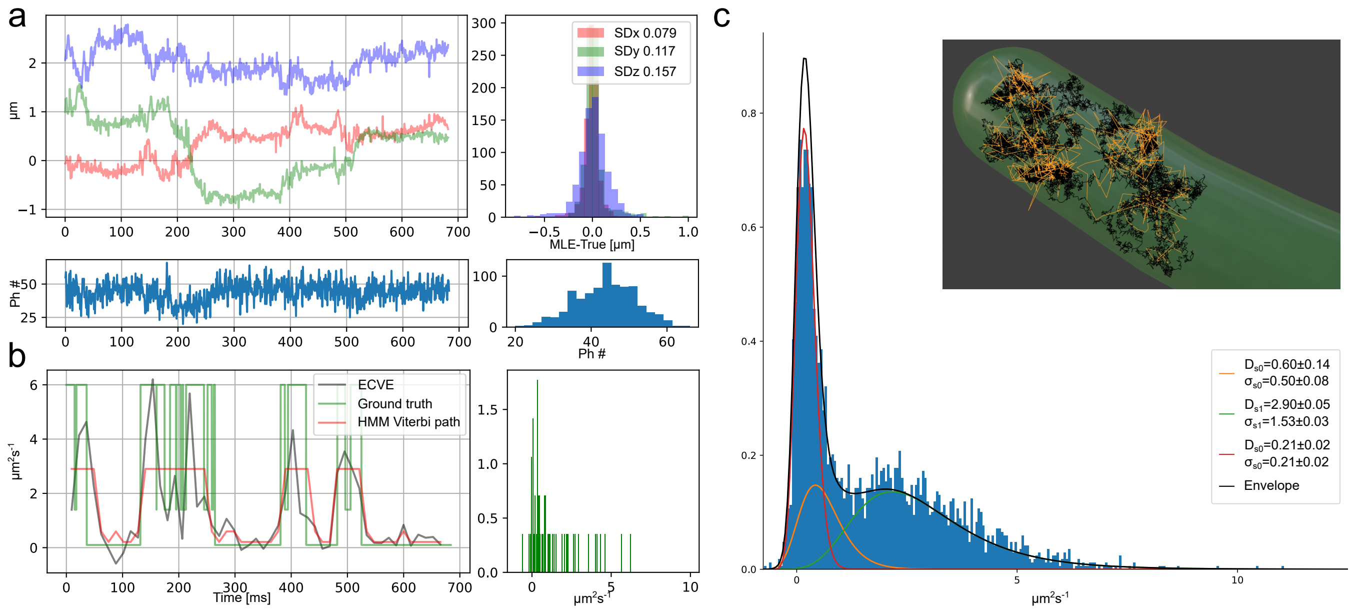

Fig. 16. Example trajectory with HMM analysis of all 100 simulated trajectories with a shot pattern with a hole at the center. **a**, upper left; example trajectory with x (red) y (green) z (blue) estimated position coordinates with corresponding photon counts (bottom). Upper right; the deviation away from ground truth with the SD of the error distribution in the legend, (bottom) is the photon count distribution. **b**, left; diffusion point estimation (gray) along the trajectory in **a**, ground truth diffusion is in green, and the HMM prediction in red. Right; the distribution of estimated diffusions over the trajectory in **a**. **c**, distribution of all diffusion estimates from 100 trajectories. The 3 state HMM model prediction of the emission distributions are plotted on top of the histogram, the inverse-gamma mean and SD with standard error of each emission distribution is reported in the legend. Inset, 3D representation of the cell volume with ground truth trajectory in black and the estimated path from **a** in yellow. Source data are provided as a Source Data file.

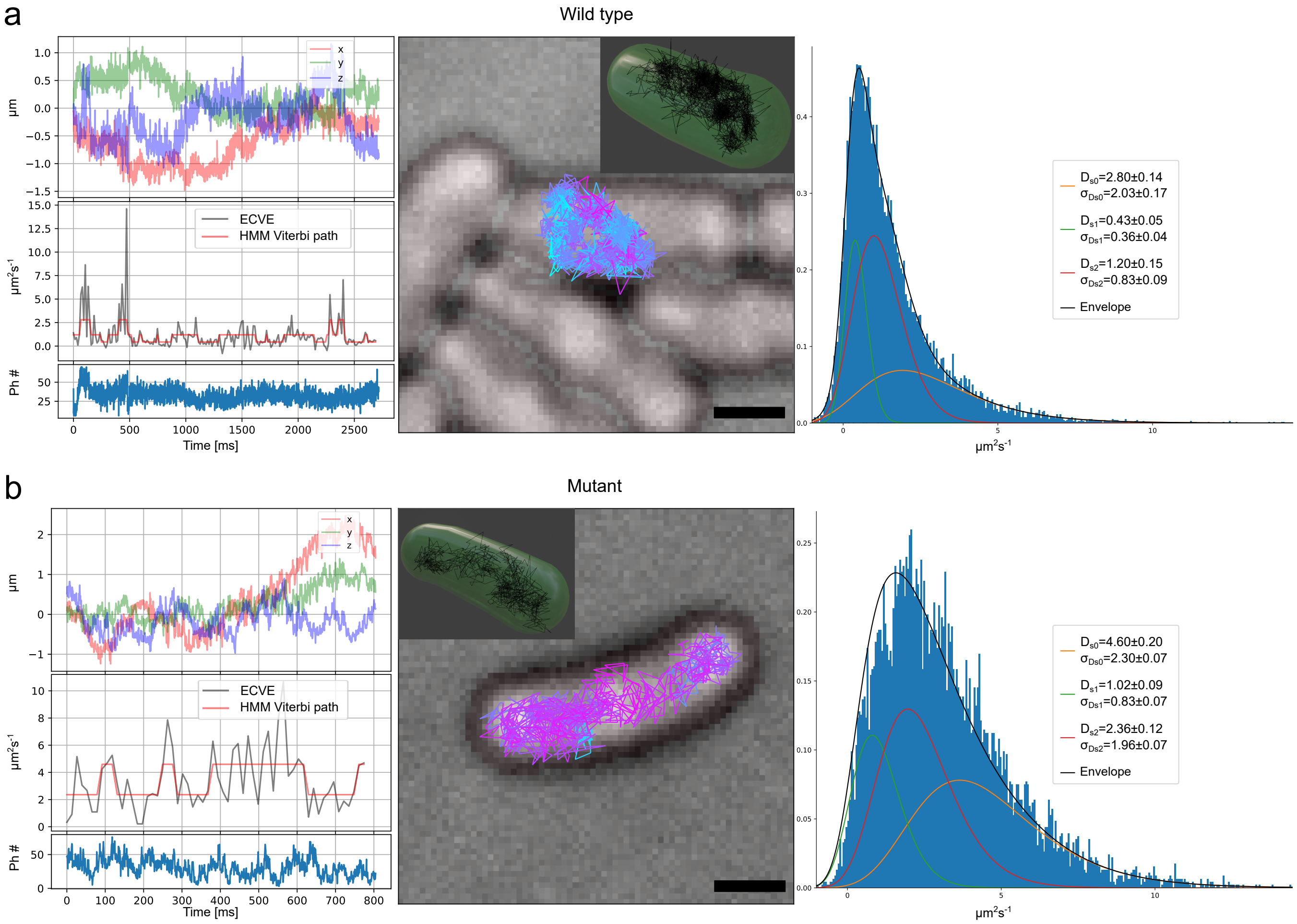

Fig. 17. **Tracking TF in WT and mutant E. Coli cells with a laser pattern with a hole at the center.** For each sample type, wild-type **a** and mutant **b**, an example trajectory (left) is shown together with the distribution of the accumulated diffusion histograms over all measured trajectories of each type (right). Each horizontal block shows: center image, widefield cell image (black bar $1\mu m$) with estimated trajectory color coded on diffusion estimation. Inset is the 3D trajectory with a cell membrane representation. Left plots, for the same trajectory shown in the center, are from the top; position estimation, point wise diffusion estimation (gray) with HMM Viterbi path (red) and photon counts in the bottom plot. Right plot; histograms over all diffusion estimates acquired in each measurement together with the HMM emission distributions. In the histogram legend are the found mean diffusion values and distribution SD with s.e esstimated by bootstraping 100 iterations over the trajectories. For the wild-type 319 trajectories were acquired from 85 cells. And for the mutant 565 trajectories were acquired from 53 cells. Source data are provided as a Source Data file.

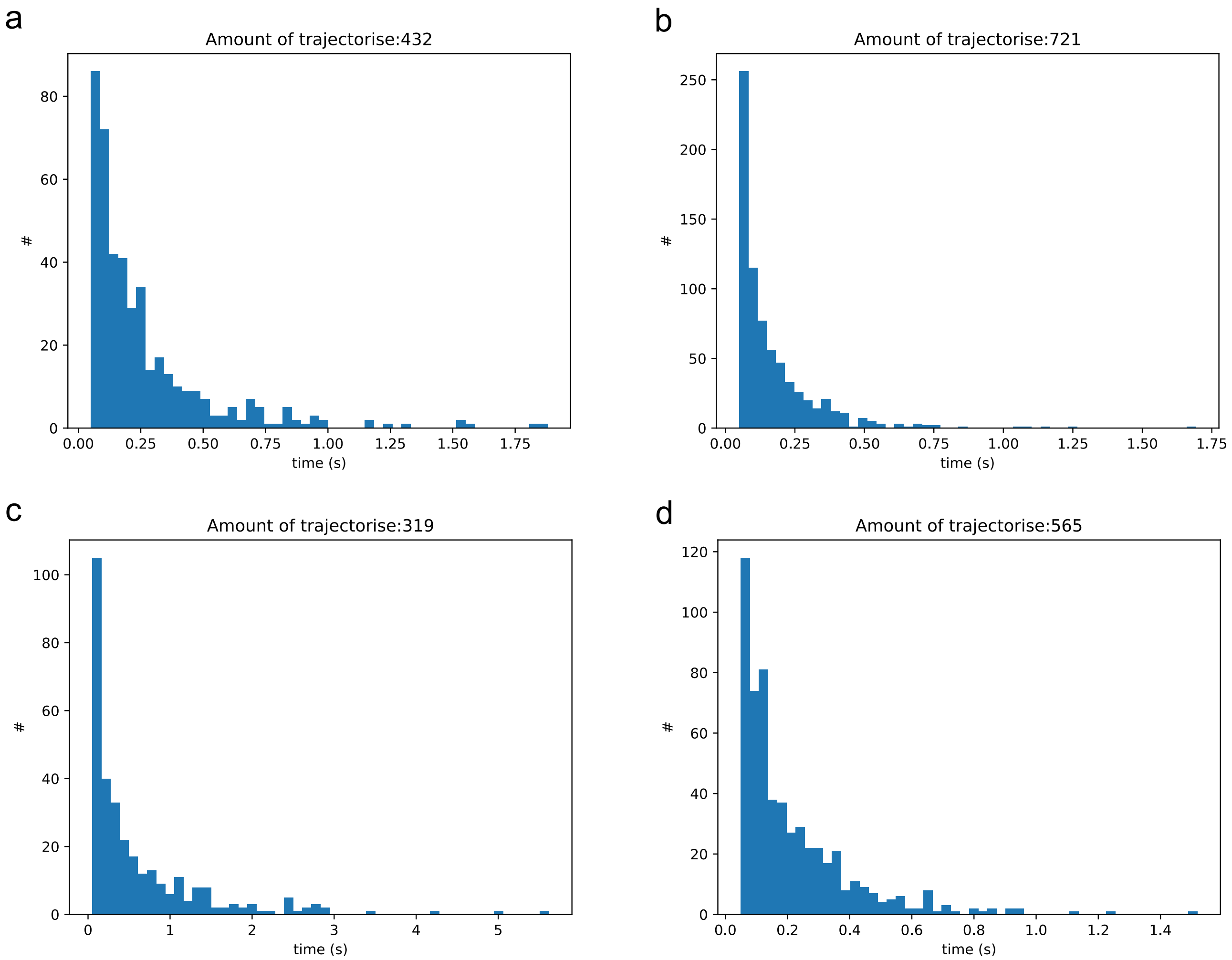

Fig. 18. **Trajectory length distributions**. **a**,**b**, shows the experiment presented in the main article, with **a** as the wild-type distribution and **b** is for the mutant. **c**, **d**, are for the experiment when removing the center shot which is presented in Supplementary Note 8, with **c** as the wild-type distribution and **d** is for the mutant. Source data are provided as a Source Data file.

I
Supplementary Note 9

The trajectory length distributions are found in Fig. 18. To avoid losing tracking events we run the real time tracking with a release threshold that occasionally is tracking background. In the post-processing, we apply an HMM on the photon counts with a running mean to identify bleaching steps. These bleaching steps are used to truncate trajectories and isolate single molecule tracking events.

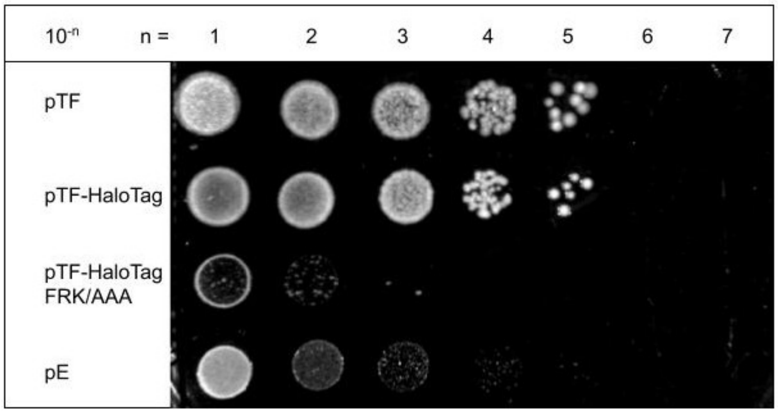

Fig. 19. **In vivo functionality of TF-HaloTag and mutant TF-HaloTag.** Viability of heat-sensitive MG1655Δ*tig::kan*Δ*dnaKJ::cm* supplemented with TF variants expressed from pQE30lacIq plasmids induced with 50 µM IPTG at 37°C after 24 h. TF variants from top to bottom: wild-type TF, TF-HaloTag, TF-HaloTag FRK/AAA mutant, empty plasmid. The experiment was performed in three independent replicates, with the same result each time.

J
Supplementary Note 10

A. *Bead sample*

Agarose was dissolved in DI to make 4% gel. After heating the gel to $80°C$, $20nm$ TetraSpeck beads were added to a final concentration of $500pM$. The bead-gel mixture was poured to form a pad between a clean glass slide and quartz coverslip.

B. *Assessment of TF-HaloTag in vivo function*

In order to ensure biological functionality of TF-HaloTag, an in vivo growth assay was performed. Due to the functional redundancy in the chaperone network, a single *tig* knockout is not lethal, as other chaperones compensate for the loss of TF. However, knocking out DnaK and its co-chaperone DnaJ along with TF results in heat sensitive cells [7]. A triple knockout strain, MG1655Δ*tig::kan*Δ*dnaKJ::cm*, was constructed by replacing *dnaKJ* with a chloramphenicol resistance cassette using λ Red assisted recombineering with primers dnaKJdel_F and dnaKJdel_R and the pKD3 plasmid (Addgene #45604) as template. This genetic replacement was introduced into MG1655Δ*tig::kan* by P1 phage transduction, creating the triple deletion strain. Cells were electroporated with pQE30lacIq plasmids encoding either untagged TF, TF-HaloTag, FRK/AAA mutant TF-HaloTag, or the empty plasmid. The plasmid encoding only TF was constructed using PCR with the pQE30lacIq_tig_halotag plasmid as template and primers pQE_HaloDel_F and pQE_HaloDel_R (Supplementary Table 2). The different strains were grown overnight at 30°C, normalized to the same optical density and serially diluted from 10^{-1} to 10^{-7}. 5 µl of the dilutions were spotted on LA plates containing 100 µg/ml ampicillin and 50 µM IPTG followed by incubation at 37°C for 24 hours to assess the heat sensitivity of each strain, see Fig. 19. Cells supplemented with the empty plasmid or the TF-HaloTag mutant are not viable, showing that, if TF does not exist or is disabled in ribosome-binding, it cannot compensate for the loss of DnaK and DnaJ. In contrast, cells supplemented with TF-HaloTag or TF alone were equally competent in compensating for DnaKJ loss, suggesting that HaloTag does not prevent the *in vivo* function of TF. It should be noted, however, that at lower IPTG level, TF-HaloTag is slightly worse than wt TF in compensating, whereas at higher IPTG level it is better than the wt TF (the mutant TF-HaloTag can never compensate). This does not affect our conclusions regarding the ribosome binding function of TF-HaloTag, and hence also not the results from our tracking experiments, but is something we are currently investigating further in a follow-up study regarding TF's chaperone function.

K
SUPPLEMENTARY TABLES

TABLE 1
OPTICAL COMPONENTS. LIST OF COMPONENTS USED IN FIG.20.

Component	Vendor
Laser	Coherent Verdi 532nm
Pockel cell	New Focus 4102NF
SLM	Hamamatsu LCOS-SLM X10468
EOD	Conoptics 311A
Tip/tilt mirror	PiezoSystem jena PSH 10/2 SG with d-drive
SPC-APD	Excelitas SPCM-AQRH
EMCCD	Andor iXon Ultra 897
Objective	Nikon Apo Lambda 100x 1.45Na
Stage	Thorlabs NanoMax stage
Dichroic mirror	Semrock FF552-Di02
Emission filter	Semrock FF01-585-40
Excitation filter	Semrock FF01-770/SP-25 and FF01-532-18
Pinhole	200μmThorlabs
CMOS camera	Allied Vision Stingray
Lenses	Thorlabs Achromatic Doublets

TABLE 2
OLIGONUCLEOTIDE SEQUENCES USED FOR CLONING.

Name	Sequence, 5´-3´
dnaKJdel_F	acaaccacatgatgaccgaatatatagtggagacgtttaggggaattagccatggtccat
dnaKJdel_R	tttttacccaggcctgcccacgggcaggcttttggggagggtgtaggctggagctgcttc
pQE_GAtig_F	agctgatgaaccagcaggcgggcgcagaaatcggtactggctttccat
pQE_GAtig_R	gtttcaactgaaacttgcatagttaatttctcctctttaatgaattctgtgtgaaattg
pQE_HaloDel_F	ttaattagctgagcttggactcc
pQE_HaloDel_R	ttacgcctgctggttcatcagct
tig_del_F	taagagttgaccgagcactgtgatttttttgaggtaacaagatgggaattagccatggtcc
tig_del_R	acgggcctttgtgcgaatttagcgcgttatgctgcgtaaagtgtaggctggagctgcttc
tig_GA_F	ttaaagaggagaaattaactatgcaagtttcagttgaaaccactcaag
tig_GA_R	aagccagtaccgatttctgcgcccgcctgctggttcatcagc
tig_mut44_46_F	gcggccgcaggcaaagtgccaatgaatatcgtt
tig_mut44_46_R	gccgtcaatacgtactttttttcgca

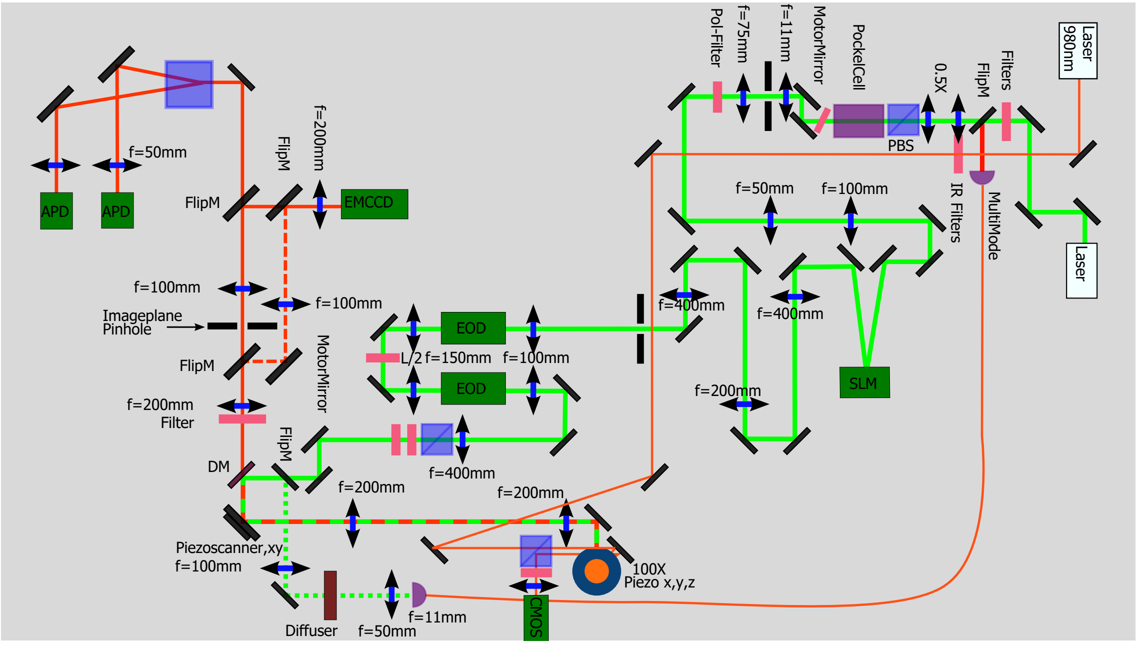

Fig. 20. **Detailed view of optics.** Green beam path represents the excitation and the red path is the emission path. Orange path represents the total internal reflection focus system.

L
Supplementary Figures

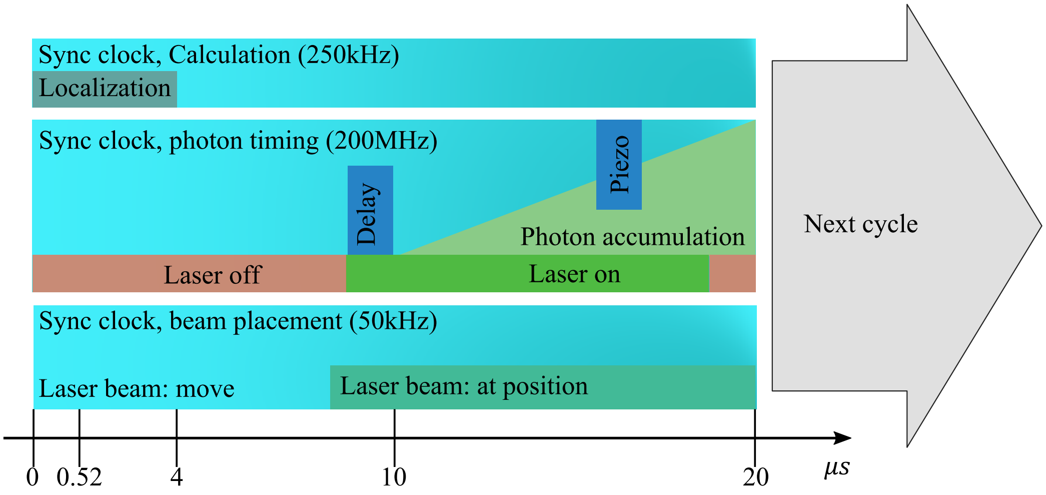

Fig. 21. **FPGA timing configuration.** This illustrates one measurement cycle. The upper loop calculates a centroid estimation and also bundles the data to be sent to the computer. The middle loop handles timing of the laser on/off, measurement delay, photon accumulation and sampling of piezo stage position. The final loop initiates each new beam movement and triggers the middle loop.

References

1. Berglund, A. J. Statistics of camera-based single-particle tracking. *Physical Review E - Statistical, Nonlinear, and Soft Matter Physics* **82,** 1–8. ISSN: 15393755 (2010).
2. Michalet, X. & Berglund, A. J. Optimal diffusion coefficient estimation in single-particle tracking. *Physical Review E - Statistical, Nonlinear, and Soft Matter Physics* **85,** 1–14. ISSN: 15393755 (2012).
3. Shuang, B. *et al.* Improved analysis for determining diffusion coefficients from short, single-molecule trajectories with photoblinking. *Langmuir* **29,** 228–234. ISSN: 07437463 (2013).
4. Vestergaard, C. L., Blainey, P. C. & Flyvbjerg, H. Optimal estimation of diffusion coefficients from single-particle trajectories. *Physical Review E - Statistical, Nonlinear, and Soft Matter Physics* **89.** ISSN: 15502376 (2014).
5. Allgower, E. L. Exact inverses of certain band matrices. *Numerische Mathematik* **21,** 279–284. ISSN: 09453245 (1973).
6. Schreiber, J. pomegranate: Fast and flexible probabilistic modeling in python. *Journal of Machine Learning Research* **18,** 1–6. ISSN: 15337928. arXiv: 1711.00137 (2018).
7. Deuerling, E., Schulze-Specking, A., Tomoyasu, T., Mogk, A. & Bukau, B. Trigger factor and DnaK cooperate in folding of newly synthesized proteins. *Nature* **400,** 693–696. ISSN: 00280836 (1999).
