# Supplementary figures and images for: Real-time single-molecule 3D tracking in E. coli based on cross-entropy minimization
Source: Nat Commun. 2023 Mar 11;14:1336. doi: 10.1038/s41467-023-36879-1 (PMC10008558; doi:10.1038/s41467-023-36879-1)

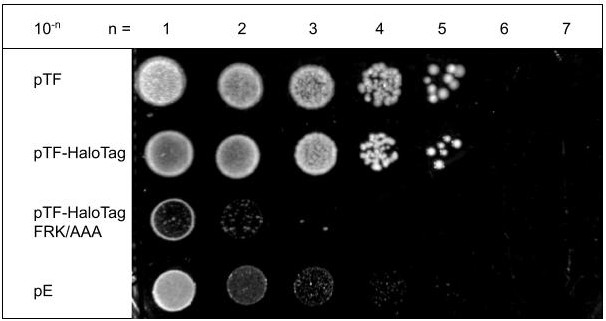

Supplement: Supplementary file 3 — Source Data [file 41467_2023_36879_MOESM3_ESM.zip › SourceData/Sup_Fig_19_Gel/221121_Shadowfax_batch_A.jpg]
